# Ultra-high-Q free-space coupling to microtoroid resonators

**DOI:** 10.1038/s41377-024-01418-0

**Published:** 2024-03-15

**Authors:** Sartanee Suebka, Euan McLeod, Judith Su

**Affiliations:** 1 Wyant College of Optical Sciences, University of Arizona, Tucson, AZ USA; 2 Department of Biomedical Engineering, University of Arizona, Tucson, AZ USA

**Keywords:** Imaging and sensing, Integrated optics

## Abstract

Whispering gallery mode (WGM) microtoroid resonators are one of the most sensitive biochemical sensors in existence, capable of detecting single molecules. The main barrier for translating these devices out of the laboratory is that light is evanescently coupled into these devices though a tapered optical fiber. This hinders translation of these devices as the taper is fragile, suffers from mechanical vibration, and requires precise positioning. Here, we eliminate the need for an optical fiber by coupling light into and out from a toroid via free-space coupling and monitoring the scattered resonant light. A single long working distance objective lens combined with a digital micromirror device (DMD) was used for light injection, scattered light collection, and imaging. We obtain Q-factors as high as $1.6\times {10}^{8}$ with this approach. Electromagnetically induced transparency (EIT)-like and Fano resonances were observed in a single cavity due to indirect coupling in free space. This enables improved sensing sensitivity. The large effective coupling area (~10 μm in diameter for numerical aperture = 0.14) removes the need for precise positioning. Sensing performance was verified by combining the system with the frequency locked whispering evanescent resonator (FLOWER) approach to perform temperature sensing experiments. A thermal nonlinear optical effect was examined by tracking the resonance through FLOWER while adjusting the input power. We believe that this work will be a foundation for expanding the implementation of WGM microtoroid resonators to real-world applications.

## Abstract (web summary)

We demonstrate free-space coupling to microtoroids using one objective to excite and collect scattered resonant light. We achieve Q-factors > 10^8^ and perform sensing with an SNR > 26 dB.

## Graphical abstract

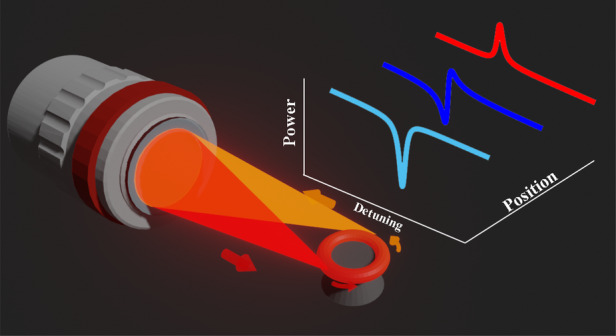

## Introduction

Whispering gallery mode (WGM) microtoroid resonators are one of the most sensitive sensors in existence due to their long photon confinement time (~10 ns)^1^, which results in a Q-factor in excess of 100 million^2^. This enables repeated interaction of light with target analytes. The high sensitivity, rapid response^3^, and label-free nature of WGM resonators have enabled many biochemical applications, including protein detection^4–6^, drug screening^7^, ovarian cancer screening^8^, exosome detection^9^, and early detection of hazardous gases^1^. FLOWER (frequency-locked optical whispering evanescent resonator) combines WGM devices with balanced detection and data processing^4,10^ to detect attomolar protein concentrations in a time scale of seconds^3,4^ and hazardous gases at part-per-trillion concentrations^11^.

Compared to other WGM resonators, microtoroids exhibit notable advantages in biochemical sensing applications due to Q-factor, material compatibility, and evanescent field interaction with analytes. Microtoroids have a higher Q-factor among WGM resonators of the same size. Here we show Q-factors as high as 100 million with 100 μm diameter devices. Other resonator geometries can only achieve a similar Q-factor by greatly increasing the device size^12^ since the radiative losses (similar to bending losses in a waveguide) vanish exponentially^13–15^. For example, Q-factors of hundreds of millions in a microring^16^ required a diameter of 23.6 mm. Compact resonators can be more densely integrated together and are more mechanically stable.

Our microtoroids are composed of silica (SiO_2_), for which robust functionalization protocols have been developed. Other materials that are highly toxic, or without easy functionalization procedures to bind receptors or targets, are not well suited for biochemical sensing applications.

Most other chip-based resonators with ultra-high Q-factor are designed to strongly confine the mode within the cavity. This strong confinement limits the interaction of the optical mode with the surrounding media. Microtoroids have a long evanescent tail that can interact with analytes because the reflow process during microtoroid fabrication generates a very smooth cavity surface. A strong interaction between the optical mode and the analyte makes the sensor more responsive (sensitive) to the analyte.

Light is typically evanescently coupled into microtoroid resonators through a tapered optical fiber hundreds of nanometers in diameter^4^. In spite of high coupling efficiency in excess of 99%^17^, these tapered fibers are fragile and suffer from vibrations due to fluid flow or air currents. Precise alignment of the fiber with the microtoroid is also needed for phase matching and, thus efficient energy transfer^18^. The drawbacks of using a tapered fiber hinder these systems from being integrated into compact and portable lab-on-chip platforms^19^ and from being multiplexed. Other coupling approaches, such as prism coupling^1^ are difficult to use with microtoroid-shape resonators. Microtoroids have advantages over other WGM sensors due to their on-chip fabrication as well as larger capture area, which enables a faster response time compared to plasmonic sensors^20^.

Integrated waveguide coupling requires high fabrication accuracy since the waveguide-resonator distance is critical. One other solution to this challenge is to integrate the waveguide after resonator fabrication, which relies on a precise nano positioner^21,22^. The free space coupling approach we show here has a higher tolerance and has the potential to be a foundation for multiplexing by directing light to an array of microtoroids through free space without the need for multiple nanopositioners. Previously, free-space coupling of light into a deformed, non-azimuthal symmetric microtoroid^23–25^ due to chaos-assisted momentum transformation^26^ was demonstrated; however, the irregular spectra and mode field distribution of these toroids limit their use in applications such as frequency comb generation^27,28^ and evanescent biosensing, which prefer a predictable response^29^. Another approach to couple light into a microtoroid from free space has been to add nano couplers randomly positioned on the microtoroid surface for indirect coupling^30^. In that approach, a fiber lens was used to deliver free-space light; however, a tapered fiber was still used to couple the light out from the cavity, and precise alignment was still required for the fiber lens.

Here, we designed a free-space coupling system for symmetric microtoroids by using a single objective lens together with a digital micromirror device (DMD). This configuration has three purposes: focusing the input light, collecting the resonant scattered light, and imaging the microtoroid. Using a single objective lens for these tasks provides a more compact system, a cheaper design, and easier alignment. A region of interest (ROI) can be selected using the DMD, which filters out some of the stray light. The advantage of imaging the resonant back-scattered light over the light transmitted in the direction of the incident beam is that there is lower background and fewer Fabry-Perot effects, which lead to higher intensity contrast^31^. This optical configuration is also compatible with other WGM resonators since they rely on the same principle. For example, monitoring microsphere resonances through scattering has been reported^32,33^. An analytic expression for the free space coupling efficiency to WGM resonators exists^34^, suggesting that maximum coupling efficiency can be enhanced by reducing the size of the Gaussian beam waist. Based on this, we compared resonant scattering power between two different objective lenses: one with a numerical aperture (NA) of 0.14 and the other with an NA of 0.42. According to the analytical solution^34^, a Gaussian beam can excite multiple azimuthal modes, denoted by mode number *m*. This creates a likelihood for the modes to overlap and generate EIT-like and Fano resonances. We examined the detuning between modes by changing the coupling strength via adjusting the beam-cavity distance. We created a coupling map to study free-space coupling position tolerance. Lastly, we demonstrated that our free space coupling system combined with FLOWER could be used for sensing applications by experimentally tracking changes in temperature.

## Results

### Resonance line shapes and system efficiency

**Fig. 1:**
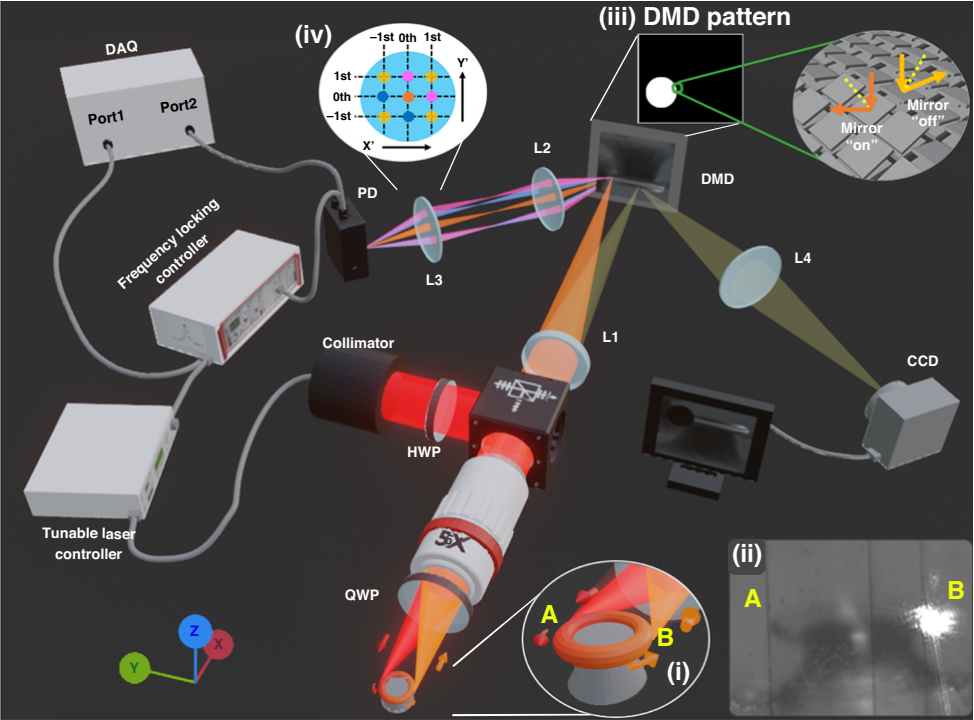
Overview of the free-space coupling system. L1: tube lens. L2 and L3 are bi-convex lenses and together form a 4f configuration to collect several diffraction orders on the photodetector (PD). L4, which is also a bi-convex lens, is the imaging lens. The yellow-brown cone indicates illumination light which comes from a ring light around the objective and not from the cavity. Inset (i) Schematic of the coupled microtoroid in free space. The laser converges at edge A to couple into the cavity as indicated by the red cone. At the resonance wavelength, the coupled wavelength is confined in the cavity. The scattering light from edge B is then collected by the same objective lens to observe the resonance wavelength as indicated by the orange cone. Inset (ii) Image from the CCD during an experiment. Vertical black lines are due to inactive pixel lines. Inset (iii) DMD pattern to select the ROI. Micromirrors in the white area are directed to the PD. The mirrors in the black area are directed to CCD for imaging. Inset (iv) Schematic of different diffraction orders on the L3 plane

**Fig. 2:**
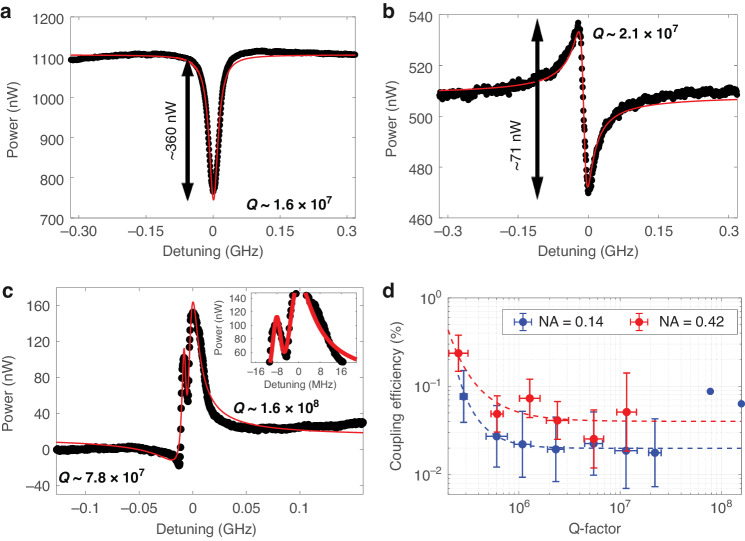
Resonance line shapes and free space coupling efficiency. **a**–**c** Resonance line shapes observed from the free-space coupling system. Black dots and solid red lines show experimental results, and their relevant curve fit depending on their shape. **a** Lorentzian line shape (fitted with Eq. (1)) **b** standard Fano line shape (fitted with Eq. (2)) **c** generalized Fano line shape (fitted with Eq. (3)). **d** Efficiency vs Q-factor for two different objective lenses. Dashed lines indicate the trend. Blue and red plots show results from NA = 0.14 and 0.42 objective lenses, respectively. The data from 140 resonance modes from 57 microtoroids was divided into ten different groups by *Q* factor in log scale. The error bars were then plotted as the standard deviation of the Q-factor and % coupling efficiency in each group, see Fig. S7 in Supplementary Note 3 for more detail

The optical system is shown in Fig. 1 and detailed in the Materials and Methods section. In this work, we observed both asymmetric Fano resonances and generalized Fano resonances in free-space coupling to a single microtoroid cavity. Two modes were simultaneously excited. These two modes have distinct quality factors, unlike the simultaneous excitation of degenerate modes in mode-splitting-based sensing^35–38^ or exceptional point sensing^39–41^. Figure 2a–c shows three different resonance line shapes observed: a Lorentzian (Fig. 2a), a standard Fano (Fig. 2b), and a generalized Fano line shape (Fig. 2c). The Lorentzian line shape is given as:

1 $${I}_{L}\left(N\right)=\frac{A}{1+{N}^{2}}+B$$

where ${\rm{{\rm N}}}=2\frac{(\nu -{\nu }_{0})}{\Gamma }$ ; *A*, *B*, Γ, *ν,* and *ν*_0_ are the amplitude, the non-resonant background, the linewidth at half maximum, the frequency, and the resonance frequency, respectively. The standard Fano line shape is the product of interference between a single resonance mode and the continuum background and is given^43^ by

2 $${I}_{{SF}}\left(N\right)=F\frac{{(N+q)}^{2}}{1+{N}^{2}}+B$$

where *q* and *F* are the asymmetry and amplitude factors, respectively. The linewidth of the standard Fano line shape is denoted by the frequency difference between the peak and dip^44^.

In the case of the generalized Fano line shape, which is a product of interaction between two modes and the continuum background^45^, the shape is given by:

3 $${I}_{{GF}}({N}_{1},{N}_{2})=B+\mathop{\sum }\limits_{i=1}^{2}{F}_{i}\frac{{({N}_{i}+{q}_{i})}^{2}}{1+{{N}_{i}}^{2}}$$

The Q-factor for each resonance is defined by the ratio of *ν*_0_ to Γ. Figure 2c shows one of the generalized Fano line shapes, where the first observed mode is a standard Fano line shape, ignoring the dip between the modes, shown in Fig. 2c inset, and the resulting quality factor is ${Q}_{1} \sim 7.8\times {10}^{7}$. The dip is present due to the existence of the 2nd mode, which has ${Q}_{2} \sim 1.6\times {10}^{8}$. See Fig. S5 in Supplementary Note 3 for more resonance line shape examples.

Depending on the phase difference between interfering modes, the different line shapes can be observed. If the interfering modes are in phase, a resonant peak would be observed. On the other hand, a resonant dip would be observed if they are out of phase. This phenomenon can be observed in tapered fiber coupling, as reported in previous work^46^. We also observe these changes in lineshape due to phase differences induced by varying the beam-cavity distance, as shown in the following subsection.

The computation of coupling efficiency relies on measuring input and output power. First, we establish that absorption losses can be neglected relative to scattering losses. Each loss mechanism can be quantified by its impact on Q-factor, according to ref. ^13^

4 $${Q}^{-1}={Q}_{{scat}}^{-1}+{Q}_{{abs}}^{-1}$$

The absorption coefficient of fused silica at 780 nm is *α* = 2 dB/km^47^, resulting in:

5 $${Q}_{{abs}}=\frac{2\pi n}{\alpha \lambda }\approx 2.5\times {10}^{10}$$

where *n* is the refractive index of fused silica. Since the experimentally measured total Q-factors are at least two orders magnitude smaller, we can conclude that absorption is negligible compared to scattering.

In steady state, the power coupled in will equal the power scattered out, and the percent coupling efficiency is

6 $$\eta =\frac{{P}_{{scat}}}{{P}_{i}}\times 100 \%$$

where *P*_*i*_ and *P*_scat_ are the free space input power after the QWP and the total scattered power from the microtoroid, respectively. We denote the fraction of *P*_scat_ captured by the limited NA of the microscope objective as $\gamma =2\pi /(2\arcsin {\rm{NA}})$ and the power directly captured by the microscope objective on-resonance as *P*_res_, which is calculated from each resonant lineshape, using the appropriate method for that lineshape. So,

7 $$\eta =\frac{\gamma {P}_{{res}}}{{P}_{i}}\times 100 \%$$

For the Lorentzian line shape, *P*_res_ is the difference between the peak/dip power and the baseline, as shown in Fig. 2a or *A* in Eq. (1).

For the standard Fano line shape, *P*_res_ is the power from the minimum dip to maximum peak, as shown in Fig. 2b. We derived *P*_res_ for the standard Fano line shape, see Supplementary Note 3:

8 $${P}_{{res},{Fano}}=\left|F\left(1+{q}^{2}\right)\right|$$

For the generalized Fano line shape, it is difficult to directly determine *P*_res_ from the plot. Here, we used parameters from the fitting equation (Eq. (3)) together with Eq. (8). Since there are two different modes, two *P*_res,Fano_ values can be obtained.

The coupling efficiency and Q-factor of 140 resonance modes from 57 microtoroids are plotted in Fig. 2d, with the raw scattering power data plotted in Fig. S7 in Supplementary Note 3. We have compared these results to theoretical predictions. To the best of our knowledge, the exact analytic solution for coupling a free space Gaussian beam to a symmetric microtoroid has not been derived. However, an analytic solution does exist for a two-dimensional circular microcavity^34^. This previous theoretical analysis based on mode overlap integrals between the free space beam and whispering gallery modes found that the ratio of intracavity power *P*_*c*_ to that of the incident Gaussian beam *P*_*i*_ cannot exceed^34^

9 $${\eta }_{\max }=\sqrt{\frac{2}{\pi }}\frac{1}{k{w}_{0}}\times 100 \%$$

where *k* is the free space wavenumber and *w*_0_ is the Gaussian beam waist. Typically *η* is relatively small, but breaking the rotational symmetry allows more modes to couple with higher efficiency, even potentially exceeding 50%^34,48,49^.

Equation (9) suggests that smaller beam waists provide greater coupling efficiency, which we confirm with our experimental measurements. Theoretically, the beam waist using a 0.42 NA objective lens is 3.9 × smaller than that using a 0.14 NA objective lens, due to the combination of the different NA and that the beam underfilled the entrance aperture for the 0.14 NA lens (see Table 1). Experimentally, we find a 2 × difference in coupling efficiency for the high Q modes in Fig. 2d, which we consider to be a reasonably good agreement.

**Table 1:** Summary of FWHM of coupling area for ×5 and ×20 objective lens

	Transverse FWHM (μm)		Spot size (μm)	Longitudinal FWHM (μm)	DOF (μm)
	*y* axis	*z* axis		*x* axis	
×5 objective lens	10.9 ± 2.5	9.4 ± 1.9	4.55	74.3	41.62
×20 objective lens	2.7	3.8	1.18	10.9	2.8

Beam spot size and depth of field (DOF) are shown for comparison

Figure 2d also shows that coupling is more efficient to modes with lower Q-factor. We performed 3D COMSOL simulations to better understand this relationship (Supplementary Note 5, Fig. S16). As in the experiment, the simulations showed higher coupling efficiency to lower Q-factor modes. We believe that the finite mesh size of the 3D COMSOL simulations effectively imparts some surface roughness to the microtoroid, which facilitates free-space coupling in these simulations.

In some other free-space coupling studies, the scattered resonant light was measured with a CCD or CMOS image sensor^32,50,51^, which is limited by the resolution, dynamic range, and frame rate. Our use of a DMD combined with a high-performance single-pixel detector here provides better resolution, controllable gain, and a fast response, while maintaining the capability for spatial imaging. The low fraction of retrieved power can be compensated by using higher input power or by increasing the photodetector exposure time. Although the coupling efficiencies are <0.1%, they are more than sufficient to obtain strong resonance peaks with high SNR (SNR ≥26 dB at *Q*~10^7^), which is all that is necessary for sensing experiments. Here, SNR is the ratio of *P*_*res*_ to noise in RMS. The maximum extinction ratio, calculated by eq. (S10) in Supplementary Note 2, is ~3.8 dB at $Q \sim {10}^{6}$.

We find that free-space excitation is particularly conducive to accessing a wide range of cavity modes. This is due to the nature of the focused Gaussian beam spot, which can couple to multiple azimuthal modes^34^. Consequently, it is easy to find overlapping modes that produce EIT-like (Fig. S5e, f) and Fano resonances. Generally, resonances in a single cavity have a Lorentzian line shape. The ability to access ubiquitous Fano resonances with a steeper slopes than Lorentzian line shapes enables improved sensitivity in sensing applications^52^ such as biological sensing or harmful gas detection^53–55^. Optical modes in the microtoroid are quasi-TE or quasi-TM modes^56–58^. Therefore, the resonance line shape can be adjusted by changing the polarization of the incident light^56^. The interference between modes leads to different lineshapes depending on the phase difference between modes. This phenomenon can be observed by changing the beam-cavity distance. Consequently, the resonant peak can convert to a Fano lineshape and to a resonant dip due to the change of phase difference. In the next section, we report how the line shape can be modified by changing the beam-cavity coupling distance.

### Effect of coupling distance and coupling stability

**Fig. 3:**
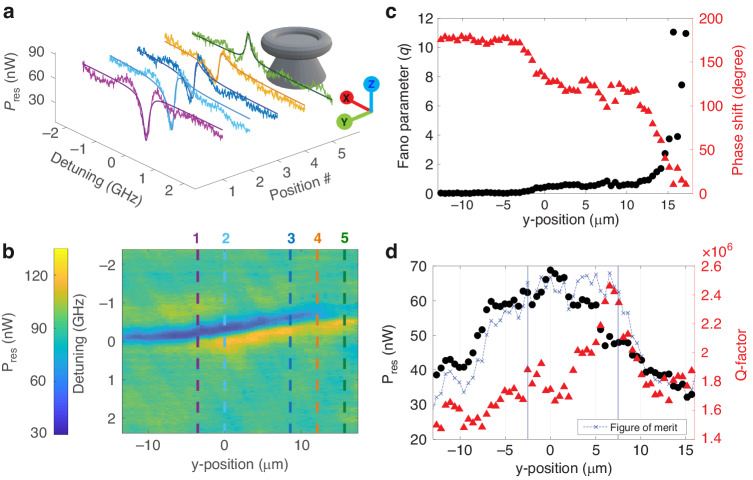
Free-space coupling map. The ×5 objective lens (NA = 0.14) was used for all panels. **a** Resonance curve at different microtoroid position numbers, which correspond to the lines indicated in **b**. **b** Color indicates the resonance power. The *y* position is defined to be zero at the highest resonance power calculated from Eq. (8). Positive y-positions mean decreased beam-cavity distance. **c** Fano parameter and phase shift vs *y* position. **d** Resonance power calculated from Eq. (8) and Q-factor calculated from linewidth obtained from Eq. (2) vs y-position. Dashed blue line indicates the figure of merit level (a.u.). *y* positions in the range of −2.5 to 7.5 μm provide maximum figure of merit

By changing the beam-cavity distance, the Fano line shape can be modified since a phase difference between the resonance mode and continuum mode is introduced. To explore this, we performed experiments in which the laser spot position was fixed and only the microtoroid was moved via a nanopositioner. Figure 3a, b show how the resonance line shapes change when the beam-cavity distance changes by moving the microtoroid along the *y* axis. At position 1, the resonance curve closely resembles a Lorentzian dip line shape. At later positions, Fano line shapes with different asymmetric profiles are observed. More line shapes are shown in Supplementary Movie S1. The Fano parameter *q*, which characterizes the asymmetry of the Fano profile and relates to the coupling strength between resonance modes and the continuum state, is the cotangent of the phase shift between two modes^42^, which varies with the beam-cavity distance, as shown in Fig. 3c. In agreement with the analytic solution for circular cavities^34^, the phase shift can be varied greatly in the over coupling regime ($y \,>\, 0$ μm), while there is only small perturbation in phase in the under coupling regime ($y \,<\, 0$ μm).

As shown in Fig. 3d, maximum Q-factor is in the over-coupling regime, not at the critical coupling condition. There is a tradeoff between resonant power and Q-factor near this range. To quantify this tradeoff, we defined the figure of merit (FoM) as ref. ^59^

10 $${FoM}=Q\times {P}_{{res}}$$

where *Q* is quality factor. The FoM for different beam-cavity distances is shown as a dashed blue line in Fig. 3d. The best FoM lies in the range of $y\in [-\mathrm{2.5,7.5}]$ μm. More resonance line shape transitions as a function of the beam-cavity distance are shown in Supplementary Note 4 (Figs. S8, S9).

**Fig. 4:**
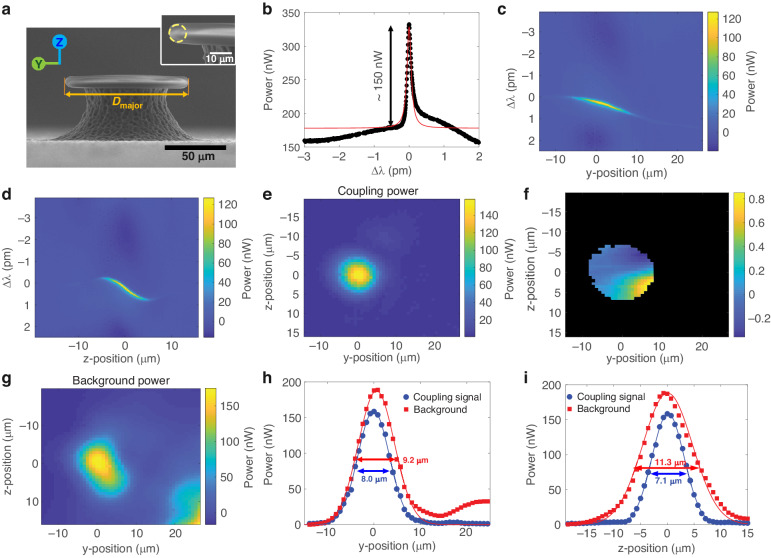
Free-space coupling map. The ×5 objective lens (NA = 0.14) was used for all panels. **a** SEM image of a microtoroid. The yellow circle diameter is the minor diameter. **b** Resonance curve at $\left(y,z\right)=\left(\mathrm{0,0}\right)$, which is the position with the highest power. **c**, **d** Spectrograms of light scattering out of the microtoroid, when scanned along the *y* axis (**c**) and the *z* axis (**d**). *Δλ* represents the detuning wavelength. **e** Resonance power map in the YZ plane (**f**) Resonance wavelength shift map in the YZ plane. Only data with coupling powers higher than 0.06 times the max coupling power are plotted in color. **g** Background map in the YZ plane. **h**, **i** Power along the *y* axis at z = 0 (**h**) and along the *z* axis at *y* = 0 (**i**). Solid lines show the fits to a Gaussian equation for determining the FWHM

The possibility of losing coupling by minor tapered fiber movements and mechanical vibration is one of the most problematic issues for the conventional tapered fiber coupling system. Here, we investigated the coupling area and how the position of the toroid affects the resonance by scanning the microtoroid position in 2 dimensions. Figure 4a is an SEM image of the microtoroid used for scanning with a major diameter (*D*_major_) of ~100 μm and minor diameter (*D*_minor_) of ~8 μm. The input laser was focused on the left-hand edge of the microtoroid as shown in Fig. 4a. The resonance curve is shown in Fig. 4b.

First, the microtoroid is scanned along the *y* axis and *z* axis (Fig. 4c, d). Values in the +*y* axis direction mean a smaller beam-cavity distance. Values in the +*z* axis direction mean the microtoroid was moved upward or that the input beam moved downward relative to the microtoroid. The unfiltered detuning wavelength vs. position maps are shown in Fig. S10. As shown in Fig. S10 and Fig. 4b, there is a background offset in the plot. We filter the background signal at each position and replot the coupling maps in Fig. 4c, d. Similar to what was observed in fiber coupling systems^60–62^, the resonance wavelength is sensitive to the gap size, especially in the over-coupling regime, $y \,>\, 0$ μm. It agrees with the analytic result that the resonance wavelength is detuned in the under and over coupling regimes^34^. In short, changing the coupling position can alter the coupling strength, which also modifies the effective index of the cavity mode^57^.

The microtoroid was then moved in the YZ plane to generate a 2D map. Each resonance curve from a different position was fitted with a Lorentzian equation (Eq. (1)). Each point in the coupling power map in Fig. 4e is the power from the baseline to the resonance peak, which is parameter *A* from Eq. (1). The position that has the highest power was defined to be the origin $\left(y,z\right)=(\mathrm{0,0})$. The resonance wavelength shifts as a function of microtoroid position are shown in Fig. 4f. By changing the position, the maximum possible resonance wavelength shift is about 1 pm. The background power, parameter *B* from Eq. (1), shown in Fig. 4g, is the combination of stray light and power from a low Q-mode that is unresolved in this scan range. The powers along the *y* axis at *z* = 0 and the *z* axis at *y* = 0 are plotted in Fig. 4h, i. The full-width at half maximum (FWHM) of the coupling power ranges from $7.1-11.3\,{\rm{\mu }}{\rm{m}}$. If mechanical vibration amplitudes are significantly smaller than the FWHM, then they are not expected to significantly affect the sensor signal.

**Fig. 5:**
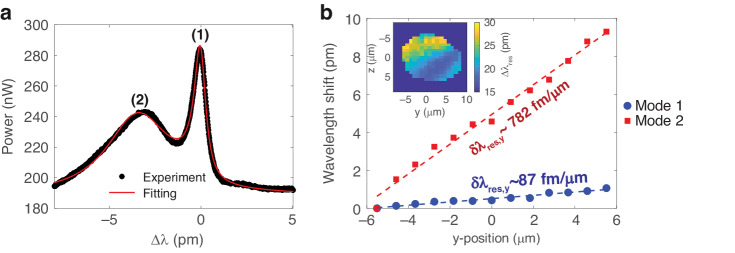
Mode crossing induced by positioning the microtoroid relative to the laser focus. **a** Resonance curve with two resonance modes fitted with Eq. (3). **b** Resonance wavelength shift along *y* axis at z = 0. (Inset) Map of the difference in resonance wavelength shift between the two modes

Changing the coupling strength between the beam and cavity by adjusting their separation causes different resonance wavelength detuning for the different modes, as shown in Fig. 5. Two-dimensional scanning in the YZ plane was performed to map the coupling power of two modes (Fig. S12 in Supplementary Note 4). The maximum power position for mode 1 was defined to be at (0,0). The resonance curve at (0,0) was fitted with Eq. (3) in Fig. 5a, and wavelength shifts was defined as zero. Both resonance modes were then tracked at each position in the YZ plane to create two resonance wavelength shift maps (${\Delta \lambda }_{{res}}$). The map of the difference of the two wavelength shifts is shown in Fig. 5b, inset. The resonance wavelength shifts along the *y* axis at *z* = 0 are plotted in Fig. 5b, which were fit to linear equations. When changing the microtoroid position, the wavelength of mode 2 shifted faster than that of mode 1. This leads to the possibility of modifying the line shape by adjusting the coupling position to get sharp Fano line shape or EIT-like (Fig. S5e, f) resonances.

**Fig. 6:**
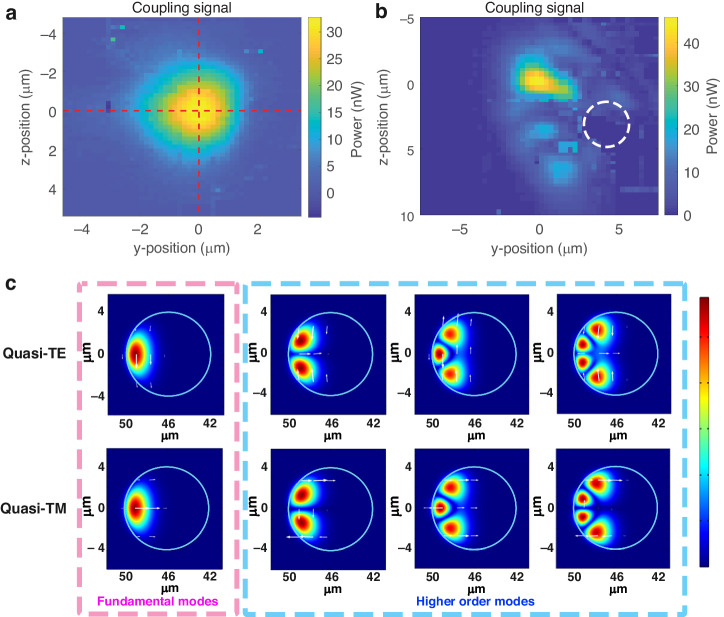
Free-space coupling map of fundamental and higher-order modes. Data were acquired using a ×20 objective lens (NA = 0.42). **a** Coupling map of the fundamental mode. Dashed red lines indicate the *y* and *z* axes. **b** Coupling map of a higher order mode. The dashed white circle shows the microtoroid cross-section corresponding to the circle in Fig. 4a, inset. The microtoroid center is on the right side. **c** Simulated electric field distribution using COMSOL. The labels along the lower axis show the distance from microtoroid’s axis of revolution. The color indicates the electric field magnitude. White arrows represent the electric field direction

Using a ×20 objective lens provides higher efficiency but a smaller coupling area due to a smaller spot size than that from the ×5 objective lens. Two coupling maps from two different modes are shown in Fig. 6a, b. The smaller spot size provides a higher resolution. A coupling map can also readily monitor the field distribution in the cavity. This type of mapping has been previously proposed using a tapered fiber^63^. Scanning in free-space has the advantage that it is easier to precisely control the beam-cavity position shift since perturbative electrostatic forces between the tapered fiber and the cavity are avoided. The coupling map in Fig. 6a shows the fundamental mode whose electric field is distributed around the equator (Fig. 6c). The coupling map in Fig. 6b has a multi-lobed distribution. The dashed white circle represents the microtoroid cross-section corresponding to the circle in Fig. 4a, inset. The microtoroid pillar is on the right-hand side of the map. We note that the circle was drawn as a guideline without an exact position measurement. The multiple lobes distributed around the microtoroid ring imply a higher order mode whose electric field has multiple lobes in the cavity (Fig. 6c). A finite element simulation was performed to show the electric field distribution in a cavity as a reference (Fig. 6c, more details in Supplementary Note 5).

**Fig. 7:**
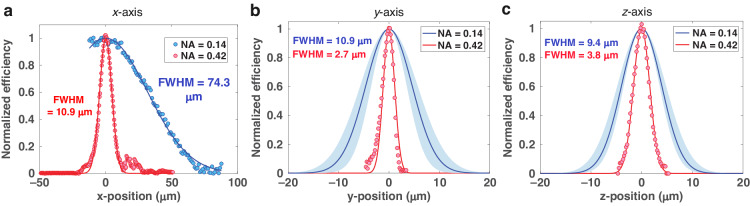
Coupling zone sizes. The normalized efficiency is shown along the **a** *x* axis, **b** *y* axis, and **c** *z* axis for a ×5 and a ×20 objective lens (NA = 0.14 and NA = 0.42). Each curve is fitted with a Gaussian equation as shown as a solid line. For NA = 0.14 in **b**, **c**, there are three sets of data. The blue solid lines show the fit to the average data. The shaded areas indicate the standard deviation from the average of experiment data (*n* = 3)

Comparisons of the coupling area FWHM between a ×5 and a ×20 objective lens, NA = 0.14 and 0.42, respectively, are shown in Fig. 7 and Table 1. The transverse coupling area FWHM is related to the beam spot size. The spot size after a Gaussian beam is focused by an objective lens is given by:

11 $$d=\left(\frac{4\lambda }{\pi }\right)\left(\frac{{f}_{{obj}}}{D}\right)$$

where *f*_obj_ and D are the effective focal length of objective lens and the input beam diameter, respectively. This equation is a paraxial approximation, which is accurate for NA < 0.9^64^. To calculate *D*, we first consider the diameter of our free-space beam, which is generated by coupling light from a single mode fiber (780-HP, Thorlabs) using a fiber bulkhead adapter and 4 cm focal length collimator lens. The output beam divergence from the single mode optical fiber (*θ*_SM_) is given by^65^:

12 $${\theta }_{{SM}}\approx \frac{0.64\lambda }{{MFD}}$$

where MFD is mode field diameter. *θ*_SM_ is half the full angular extent of the beam in radians. The collimated beam diameter (*D*_*c*_) was then calculated by:

13 $${D}_{c}=2{f}_{c}\tan {\theta}_{{SM}}\approx 0.87\,\rm{cm}$$

where *f*_*c*_ is collimator lens focal length. However, *D* is also limited by the pupil diameter (*D*_*p*_).

For the ×5 objective lens, $D={D}_{c}$ in Eq. (11), as the pupil diameter (1.12 cm) is larger than collimated beam (0.87 cm). For the ×20 objective lens, the input collimated beam overfilled the pupil (0.84 cm in diameter). Since the overfill factor is small, the spot size is approximately calculated by Eq. (11) when $D={D}_{p}$.

For the ×5 objective lens, the FWHM along the *y*- and *z* axes are 10.9 ± 2.5 μm and 9.4 ± 1.9 μm. The spot size is 4.55 μm. For the ×20 objective lens, the FWHM along the *y*- and *z* axes are 2.7 μm and 3.8 μm, respectively. The spot size is 1.18 μm. The transverse FWHM is about 2–3 times bigger than the spot size.

The FWHM along the *x* axis, which is parallel to the objective lens optical axis, is 74.3 and 10.9 μm for the ×5 and ×20 objective lenses, respectively. The depth of focus (DOF) is given by:

14 $${\rm{DOF}}=\frac{\pi {d}^{2}}{2\lambda }$$

where *d* is the focused beam spot diameter. Therefore, the estimated DOF is 41.6 μm and 2.8 μm for the ×5 and ×20 objective lens, respectively. In short, higher NA provides higher coupling efficiency, but a smaller coupling area due to a smaller spot size and short DOF. The summary of coupling area FWHM, spot size and DOF comparison is shown in Table 1.

### Temperature sensing experiment and thermal nonlinear optical effect

**Fig. 8:**
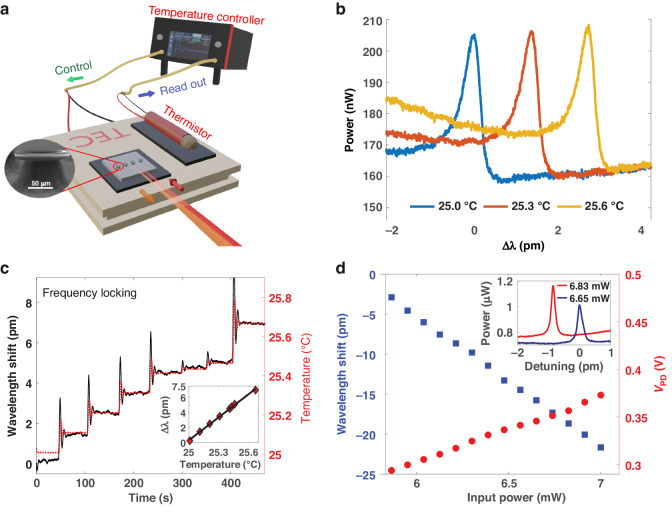
Temperature sensing experiment using free-space coupling. **a** Schematic of the experimental setup. Red and orange cones represent input and scattering light, respectively. **b** Resonance curves at different temperatures. *∆λ*, the wavelength detuning, is defined to be zero for the peak wavelength at the starting time. **c** Temperature sensing results using FLOWER. The black and red lines indicate resonance wavelength shift and temperature measured from the thermistor. (Inset) Resonance wavelength shift vs temperature. The sensor shows a strong linearity providing a slope of 10.2 pm/°C. **d** Thermal nonlinearity observation. The resonance wavelength shift and photodetector signal (*V*_*PD*_) vs free space input laser power using FLOWER. (Inset) Resonance curves with two different free space input laser powers were observed using a scanning method

To verify the application of our free-space coupling system to sensing tasks, we used the microtoroid to track small changes in temperature, using the setup shown in Fig. 8a (see also Materials and Methods). Resonance wavelength shift corresponds to temperature. A thermistor was used to independently verify the temperature. Figure 8b shows how the resonance wavelength increases with temperature. Comparisons of sensorgrams from the microtoroid and the thermistor, plotted in Fig. 8c and Fig. S17, show good agreement as the temperature and resonance wavelength shift data overlap well. The strong linearity of both methods was observed, as shown in Fig. 8c inset and Fig. S17b, confirming that our free-space coupling system can be used for sensing applications.

When tracking the resonance wavelength shift using the scanning method, the position of the resonance peak was located after each scan. There is a tradeoff between data acquisition time and resolution: shorter scanning periods lower the wavelength resolution. Also, there is another tradeoff between dynamic range and resolution. FLOWER is more efficient than scanning in terms of sampling frequency and resolution since a full scan sweep is unnecessary.

The scanning method’s resolution ($\delta {\lambda }_{{\rm{res}},{\rm{scan}}}$) can be calculated by the following equation:

15 $$\delta {\lambda }_{{\rm{res}},{\rm{scan}}}=\frac{{2f}_{{sr}}}{{f}_{{PD}}}\left(\Delta \Lambda \right)$$

where ΔΛ, ${f}_{{sr}}$ and ${f}_{{PD}}$ are the scanning wavelength range, the scan rate, and the photodetector bandwidth. Here, the system is limited by the photodetector bandwidth (3 kHz) since it is slower than DAQ bandwidth (~200 kHz). A scan rate of 10 Hz and a scan range of 7 pm were used for the experiment shown in Fig. S17. We found $\delta {\lambda }_{{\rm{res}},{\rm{scan}}} \sim 46\,\rm{fm}$ and the scanning method’s sampling frequency is 10 Hz.

Here, the tunable laser configuration is a Littman-Metcalf. The resolution is limited by how finely the piezo transducer can tune the mirror angle, which depends on the piezo voltage resolution. By using the FLOWER system, it is possible to detect wavelength shifts ($\delta {\lambda }_{{\rm{res}},{\rm{FLOWER}}}$) at sub-attometer levels^10^. The modulation frequency (FLOWER sampling frequency) used in Fig. 8c was 1 kHz. The dynamic range was limited by the laser frequency modulation range (60 GHz), which is equivalent to 120 pm at 775 nm. From this analysis, we can conclude that the FLOWER technique is superior to the scanning method in terms of resolution, sampling frequency, and dynamic range. Also, data analysis is simpler with the FLOWER technique.

The refractive index dependence on the intracavity optical intensity, a nonlinear optic effect, resulted from two different mechanisms: the photothermal effect and the Kerr effect^66^. The origin of the photothermal effect is absorption within the cavity. Consequently, intense optical modes lead to heating of the microtoroid and a change in the refractive index of material. Refractive index changes due to the Kerr effect originate from a nonlinear response to electric field.

Changes in refractive index were examined through the change of resonance wavelength when varying the free space laser power (see also Materials and Methods). As shown in Fig. 8d inset, the resonance wavelength decreases as the laser intensity increases, which indicates a corresponding decrease in refractive index, since ${\lambda }_{{eff}}=\lambda /n$ should be conserved for a given mode. The resonant scattered power, monitored through the photodetector signal (${V}_{{PD}}$) linearly increases as the input power increases. The wavelength shift from this experiment exhibits the opposite behavior from the result in Fig. 8c, where the resonance wavelength increases as the temperature increases. This behavior is observed because the TEC heats the entire chip resulting in a contribution from the thermo-elastic effect of silicon substrate^66^.

## Discussion

The use of fragile tapered fiber is a main barrier to commercializing microtoroid resonator sensors for field use. As an alternative, we introduced free-space coupling to WGM microtoroid resonators using a single long-distance objective lens by monitoring resonant scattering from the cavity. Q-factors in excess of 100 million were obtained. The system design is also compatible with other WGM mode resonators and other wavelengths as they employ the same principle. Here, the optical components were designed for use in the near-infrared waveband, as the DMD reflectivity is larger than 95% from 700 nm to 2500 nm. At other wavelengths, some components may need to be replaced to fit the application. We showed that a more tightly focused beam can increase the free-space coupling efficiency in agreement with previous theoretical predictions^34^. EIT-like and Fano resonances in a single cavity were easy to find due to multimode coupling from a Gaussian beam due to its capability of coupling to multiple azimuthal modes. The Fano line shape can be modified by adjusting the beam-cavity distance because the effective refractive indices of modes shift at different rates. The sharp Fano line shape has great potential for optical switching and enhancing biochemical sensing sensitivity^56^. We introduced a FoM to quantify the tradeoff between resonant power and Q-factor. A loosely focused beam provides lower free-space coupling efficiency, but less sensitivity to mechanical vibration due to a larger effective coupling area (~10 μm in diameter for NA = 0.14). A coupling map created by scanning the beam-cavity position can be used to monitor the electric field distribution in the cavity, which was also studied by using finite element simulation. Sensing applications were verified by combining the free-space coupling system with a frequency-locking technique called FLOWER. A thermal nonlinear optical effect was observed as the refractive index changed when the intracavity optical power changed. We believe that free-space coupling into microtoroid resonators can be used for spectroscopy and biosensing, and can become the foundation of fully on-chip WGM microtoroid resonator sensing systems or applications where optical fiber usage is infeasible, for example, in measuring the Q-factor of intracellular WGM lasers or where one does not have access to an optical spectrum analyzer.

## Materials and methods

### Microtoroid fabrication

Microtoroid resonators were fabricated as previously described^4^ using photolithography and thermal CO_2_ laser reflow. Shipley S1813 photoresist was spun coat on a 2 μm thermally grown silica (SiO_2_) layer on top of a silicon wafer (University Wafer, MA). After exposing UV light through a photomask consisting of opaque arrays of 150 μm diameter circles to the photoresist layer, 150 μm diameter circular discs were patterned on the photoresist. The exposed silica areas were etched away using a 6:1 (v/v) buffered oxide etchant. The etching stopped at the silicon layer. The remaining photoresist was removed with acetone and washed with isopropyl alcohol. Silica discs with diameter of 150 μm were left on a silicon substrate. Samples were baked at 175 °C for at least 10 mins to remove moisture. A xenon difluoride (XeF_2_) etch was performed, undercutting the silicon and creating silica microdisks. Finally, thermal reflow by a CO_2_ laser (Synrad, WA) was done to produce the finished microtoroid resonator structure, which had a major and minor diameter of ~100 μm and ~8 μm, respectively (see Fig. S1 in Supplementary Note 1).

### Optical configuration

An overview of the free-space coupling system is shown in Fig. 1. A tunable laser (Velocity^TM^ TLB-6712, Newport) with a tuning range of 765–780 nm is used. A long working distance objective lens (NA = 0.14, ×5 M Plan Apo NIR, Mitutoyo) is used for three purposes: focusing laser light, collecting scattered light, and imaging the microtoroid. A DMD (854 × 480 pixels, pixel pitch of 5.4 μm, DLP2010, Texas Instruments) separates the scattered light from the cavity into two beams: one for monitoring the resonance wavelength shift using a photodetector and the other for imaging the microtoroid. In later experiments, a higher NA objective lens (NA = 0.42, ×20 M Plan Apo, Mitutoyo) was used for comparison.

The laser, indicated by the red path, passes through the collimator, half-wave plate (HWP), polarizing beam spitter (PBS), objective lens, and quarter-wave plate (QWP) before reaching the toroid. The HWP is used for polarization rotation to maximize the output power. The QWP changes the polarization state of the scattered light so that it transmits through the PBS. Here, we define the TE and TM waves to have polarization parallel to the *z* axis and xy-plane, respectively. The light reflected light from the PBS is TE, which becomes circularly polarized after passing through the QWP. This configuration can also behave like an isolator to reduce the back reflection from the objective lens, although it is not a true isolator since there is no external magnetic field in our system. Output free-space light converges to one of the microtoroid edges (edge A shown in Fig. 1 inset (i-ii)). On-resonance wavelengths are then coupled and confined in the cavity. Some amount of the confined light in the cavity scatters out as indicated by the orange path. The objective lens collects scattered light from the opposite edge (edge B shown in Fig. 1 inset (i-ii)). Because the polarization state of the out-coupled light has been modified from that of the in-coupled light due to resonance within the microtoroid, a significant amount of light can pass through the PBS rather than being reflected back to the source. This transmitted light then passes through the tube lens (L1) and converges on the DMD. For imaging purposes, a ring light is fitted around the objective lens for illumination. The illumination scattered back from the microtoroid, shown by yellow-brown cones in Fig. 1, goes through the objective lens and is imaged on the DMD plane.

To distinguish the resonant scattered light from illumination light, custom LabVIEW software was developed to upload the DMD pattern and select an ROI. As shown in Fig. 1 inset (iii), micromirrors in the white region tilt to a +17° angle, which directs light to the photodetector (PD) (PDA100A2, THORLABS). After the DMD, light diffracts in several orders due to the grating effect of the DMD. Different diffraction orders are represented by different colors, as shown in Fig. 1 inset (iv). To reduce diffraction loss, L2 and L3 are used to form a 4f-configuration. Different diffraction orders will converge to the same point at the PD. The aperture is large enough to capture the 2nd order. For imaging, micromirrors in the back region, Fig. 1 inset (iii), tilt to a -17° angle to direct the maximum intensity order to the imaging lens (L4) whose aperture filters out the other orders. Figure 1 inset (ii) shows an image taken during an experiment. The input laser couples in at edge A. The resonant scattered light leaks from edge B.

During the experiment, a microtoroid chip is placed on a nanopositioner (P-611.3, Physik Instrumente) for alignment. The image from the CCD helps us to locate the microtoroid, position the toroid to focus the input light into it and see the resonant scattered light area. After selecting the ROI around edge B from Fig. 1 inset (ii) to filter out stray light, as mentioned above, the DMD splits the resonant scattered light and illumination into two different paths. Light in the ROI is delivered to the PD. Consequently, the ROI appears dark on the CCD, which also serves as a cross-reference to ensure that the ROI is at the desired location (see Figs. S3, S4 in Supplementary Note 2).

### Temperature sensing experiment and thermal nonlinear optical effect

The microtoroid temperature was adjusted using thermoelectric cooling (TEC) (TECF2S, THORLABS). The microtoroid chip and thermistor (GL202F9J, Littelfuse) were attached to the TEC using thermally conductive tape (Fig. 8). A temperature controller (SLICE-QTC, Vescent Photonics) generated a temperature control feedback loop by reading out the temperature from the thermistor and controlling the TEC to either heat or cool. The resonance wavelength shift corresponding to temperature was then measured by two different methods: the scanning method and the frequency locking method (FLOWER).

For the scanning method, a DAQ (PCI-4461, National Instruments) recorded the resonance curve from the PD, port 2 in Fig. 1, while the tunable laser kept scanning through various wavelengths. The resonance wavelength at each time was then extracted from each resonance curve. For the frequency locking method, we used FLOWER, which was developed in our group for aqueous biological sensing^6,9,10^. In short, a frequency-locking feedback controller reads the signal from the PD and sends out the voltage to a tunable laser controller to adjust the laser frequency to the cavity resonance. A DAQ reads the voltage from the frequency locking controller, port 1 in Fig. 1, which corresponds to the resonance wavelength shift.

The refractive index dependence on the intracavity optical intensity can be examined by monitoring the resonance scattering while adjusting the input laser power and using frequency locking (FLOWER) to stay on resonance. The advantage of FLOWER is that we can specifically deliver the power to the observed resonance, without distributing power across a wide range of wavelengths, as would be required if using the scanning method to track resonances.

### Supplementary information

Supplementary material

Movie S1
