# Supplementary material for: Ultra-high-Q free-space coupling to microtoroid resonators
Source: Light Sci Appl. 2024 Mar 15;13:75. doi: 10.1038/s41377-024-01418-0 (PMC10942989; doi:10.1038/s41377-024-01418-0)
Supplement: Supplementary file 1 — Supplementary material [file 41377_2024_1418_MOESM1_ESM.docx]

**Supplementary Information for**

Ultra-high-Q free space coupling to microtoroid resonators

**This PDF file includes:**Note 1: SEM images of microtoroids and dimensions

Note 2: Optical configuration

Note 3: Additional resonance curves and system efficiency calculation

Note 4: Additional coupling map

Note 5: Finite element simulation

1. Electric field distribution
2. Coupling efficiency

Note 6: Temperature sensing using scanning method

**Other Supporting Online Material for this manuscript includes the following:**
Movie S1

**MovieS1:**

**Title: Line shape transition as scanning beam-cavity distance changes**

**Caption:** Line shape transition vs different beam-cavity distance along the y-axis. Zero is defined as the position that provides maximum power.

**Supplementary Note 1:** SEM images of microtoroids and dimensions

**
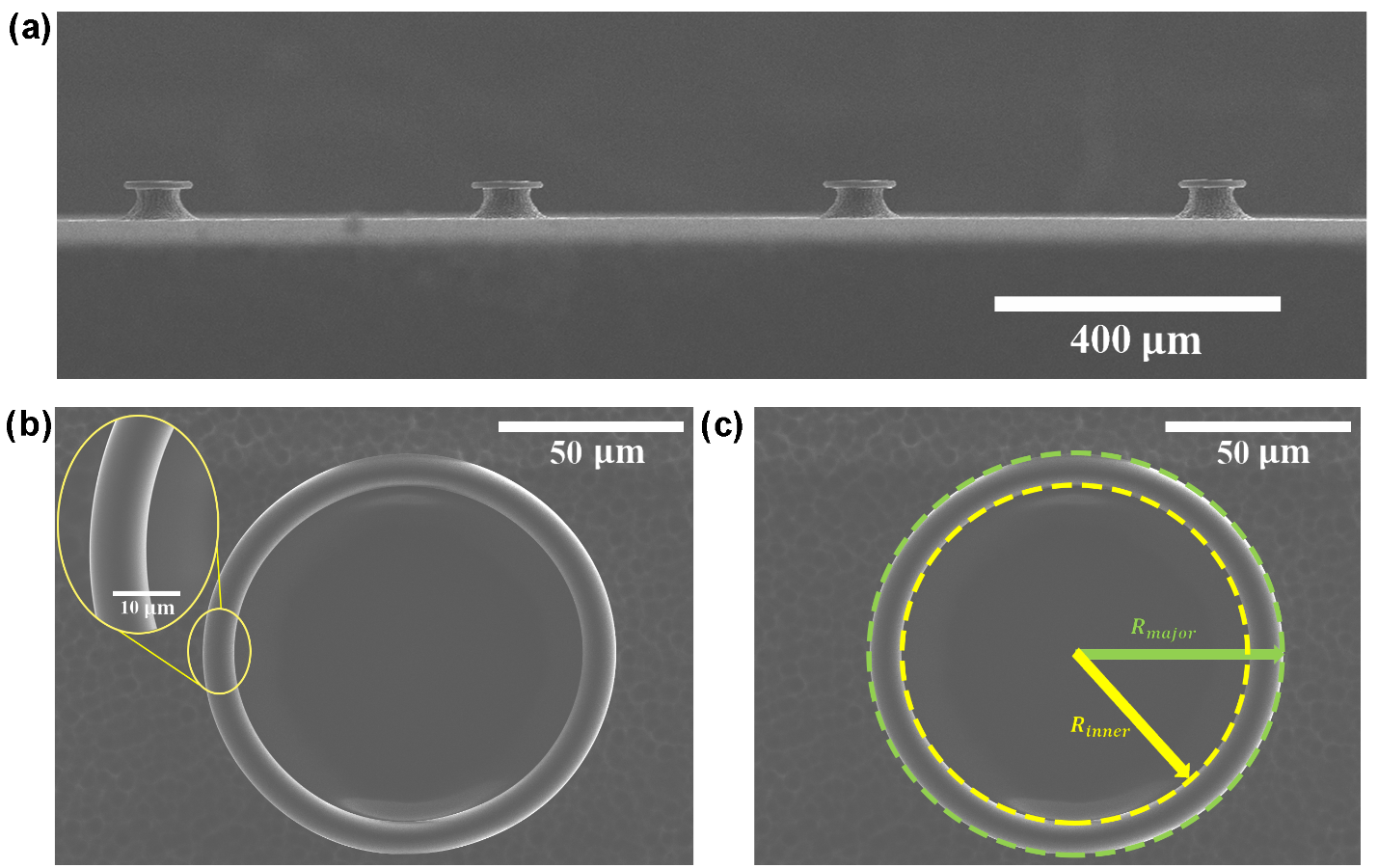
**

**Fig. S1** SEM images of microtoroid resonators. **(a)** Microtoroid array **(b-c)** Microtoroid; top view. Dashed yellow and green circles are drawn to find the major radius and minor radius.

The fabricated microtoroids’ major radii ($R_{major}$) and minor radii ($R_{minor}$) were extracted by drawing the inner circle ($R_{inner}$) and outer circle ($R_{outer}$) as shown in Fig. S1(c). $R_{major}$ and $R_{major}$ are given by:

|  | $R_{major}=R_{outer}$ | (S1) |
| --- | --- | --- |
|  | $R_{minor}=\frac{R_{outer}-R_{inner}}{2}$ | (S2) |

In these experiments, $R_{major} \sim50$ µm and $R_{minor} \sim4$ µm.

**Supplementary Note 2:** Optical configuration


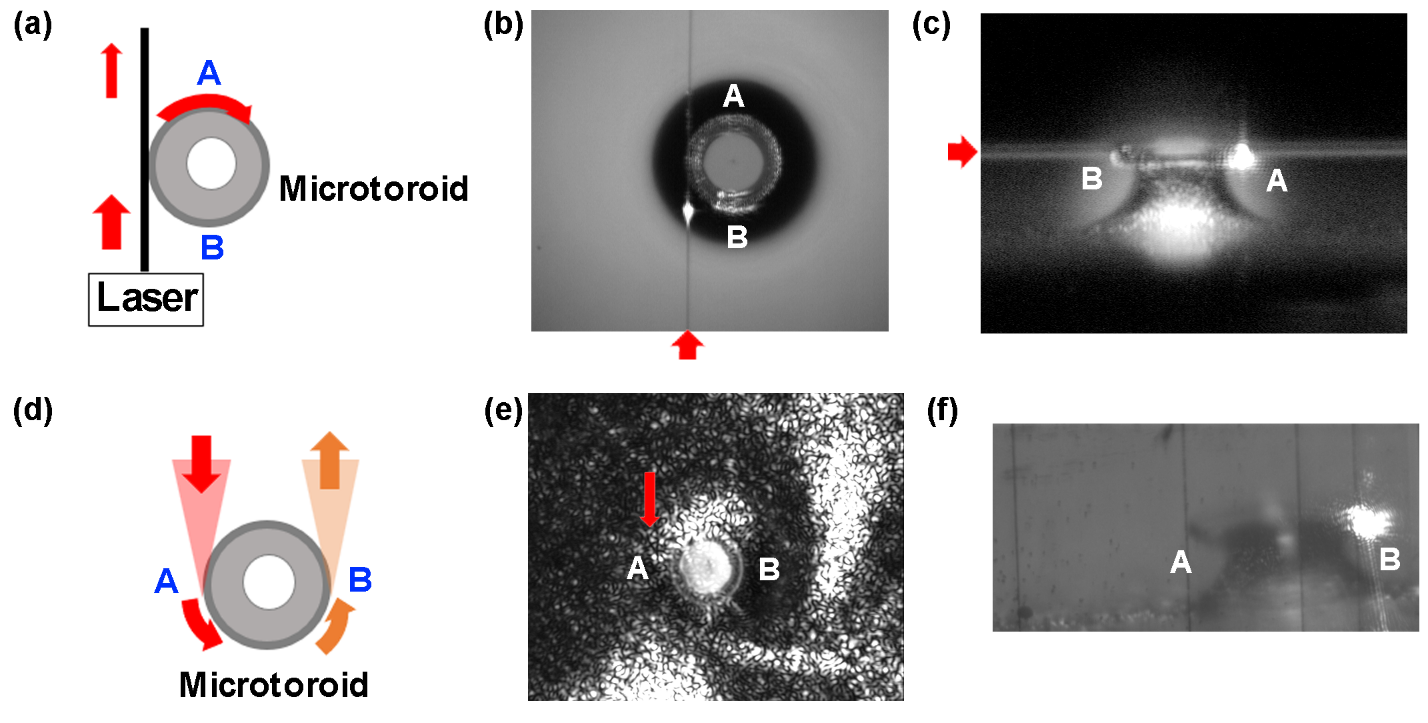


**Fig. S2** Microtoroid coupling images. **(a)** Tapered fiber coupling configuration **(b)** Microtoroid coupled with a tapered fiber, top view. The red arrow indicates the input laser. **(c)** side view of (b) **(d)** Free-space coupling configuration **(e)** Microtoroid coupled with free space light, top view. The red arrow indicates the input laser. **(f)** side view of (e) using the system in Fig. 1.

In the tapered fiber coupling system, the resonance curve is monitored through the transmitted light of the tapered fiber. It is also possible to monitor through the resonant light scattered^1^ from edge A as shown in Fig. S2(c). In this work, we instead used the configuration in Fig. S2(d). By focusing light at one edge (edge A), light at resonance couples into and circulates in the cavity. Light in the cavity scattered at the other edge (edge B) is collected by an objective lens for monitoring. Fig. S2(e) shows a top view of the free space coupling experiment. Light leaking and scattering out from the cavity are circulating in the cavity as seen as a vortex-like shape in Fig. S2(e).


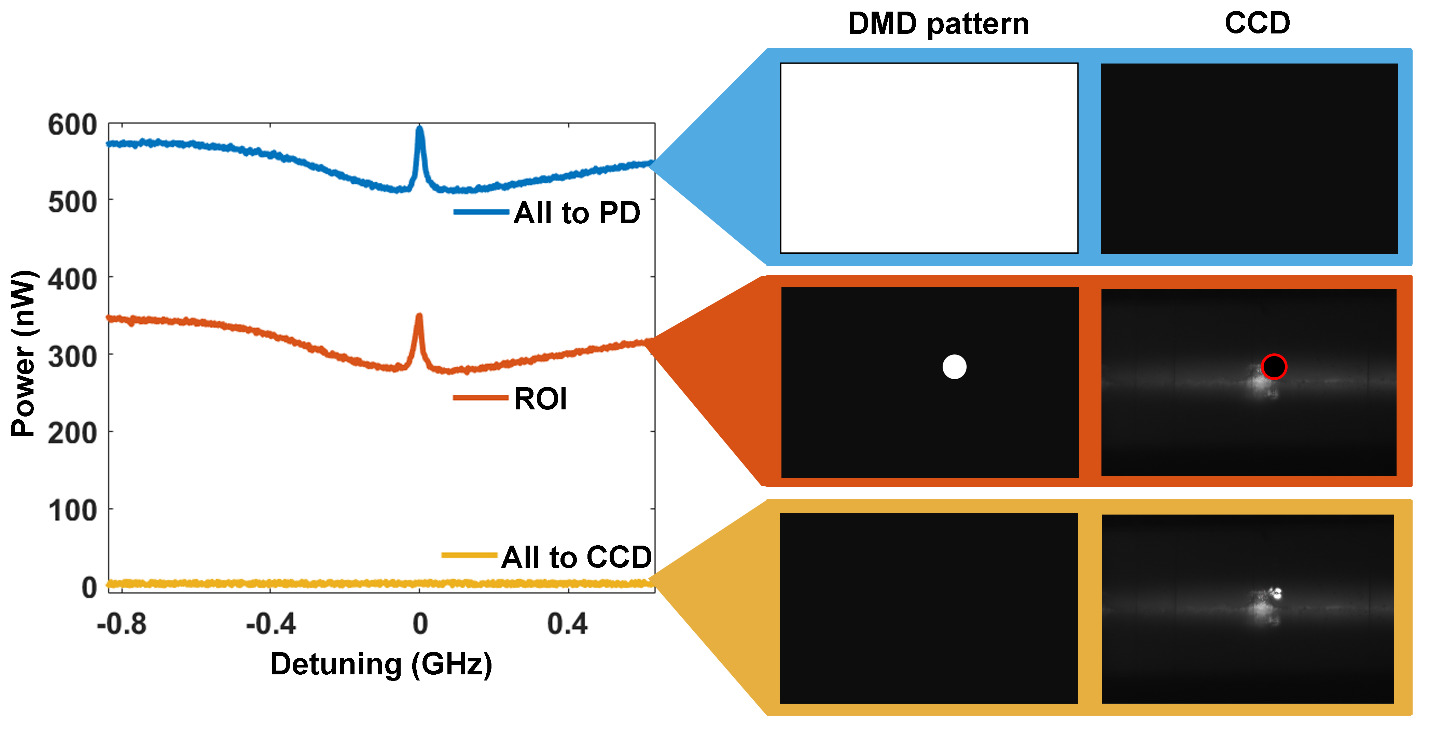


**Fig. S3** Resonance curve (left) and images from CCD (right) corresponding to DMD patterns using a 5× objective lens. The white area on the DMD indicates the area that micromirror tilts +$17^{\circ}$ to direct light to the PD. The micromirrors in the black area tilt +$17^{\circ}$ to direct light to CCD.

**
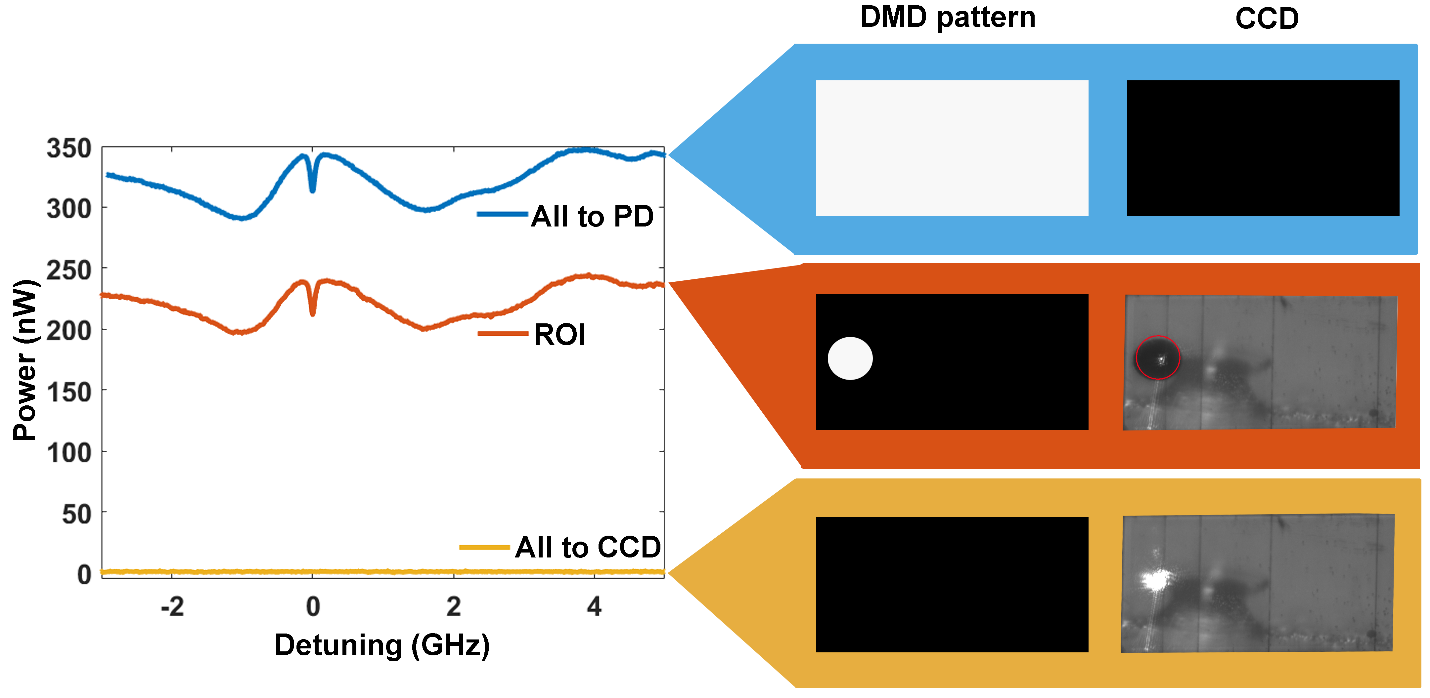
**

**Fig. S4** Resonance curve (left) and images from CCD (right) corresponding to DMD patterns using a 20× objective lens. The white area on the DMD indicates the area that micromirror tilts +$17^{\circ}$ to direct light to the PD. The micromirrors in the black area tilt +$17^{\circ}$ to direct light to the CCD.

In addition to the configuration in Fig. 1, a digital micromirror device (DMD) is used to select a region of interest (ROI) as shown in Fig. S3-Fig. S4. Black and white areas are used to separate the micromirror tilt angle. The micromirrors in the white and black areas direct incident light to the photodetector (PD) and CCD, respectively. By selecting an ROI, some amount of stray light is filtered out. The image on the CCD is utilized to verify if DMD pixels we selected corresponding to the ROI we want. The image appears dark at the areas tilted towards the PD.

**Supplementary Note 3:** Additional resonance curves and system efficiency calculation


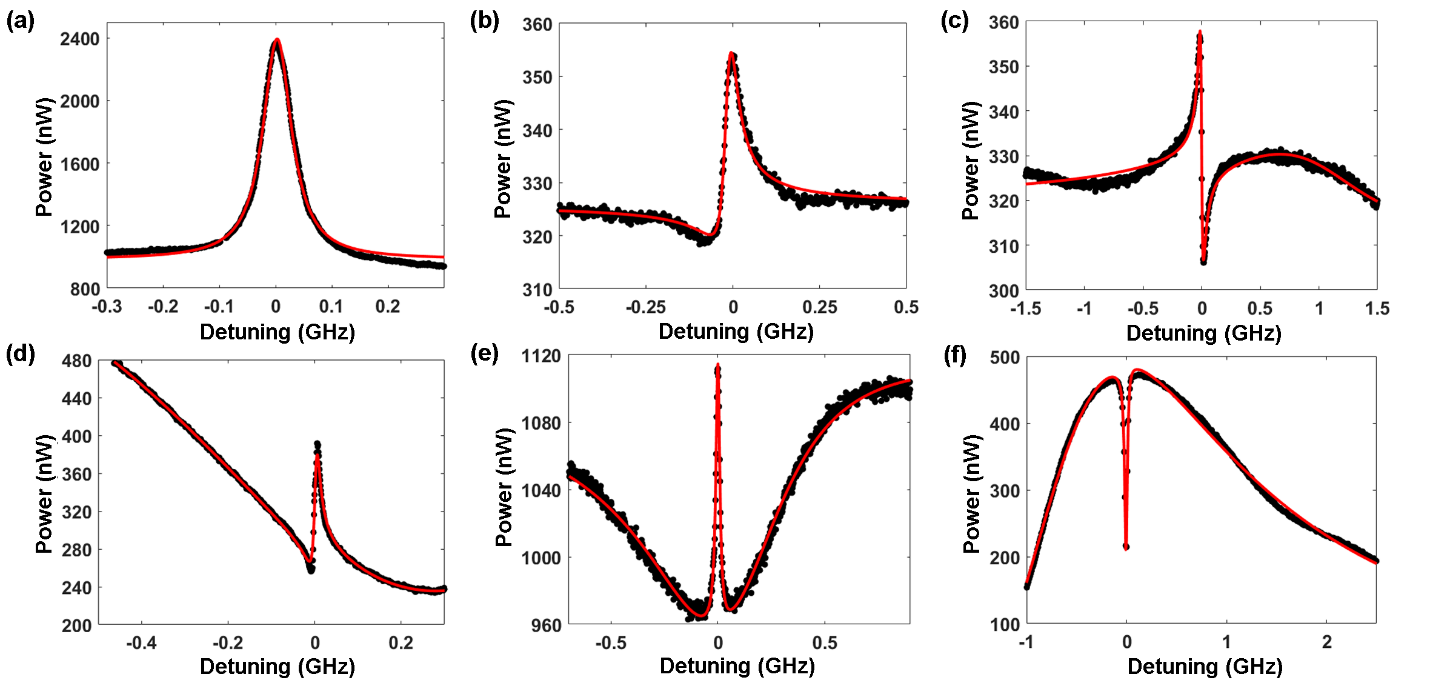


**Fig. S5** Additional resonance curves. The black dots and red lines show the experimental result and fits, respectively. **(a)** Lorentzian line shape fitted with Eq. (1) in main text. **(b)** Standard Fano line shape fitted with Eq. (2) **(c-f)** Generalized Fano line shapes fitted with Eq. (3).

Fig. S5 shows additional resonance curves beyond those already in Fig. 2(a-c). We categorized resonance curves into three types: Lorentzian, standard Fano, and generalized Fano line shapes. The Lorentzian line shape is described by Eq. (1) in main text.

To calculate system efficiency from Eqns. (6)-(7), $P_{res}$ for the Lorentzian line shape is the power from the baseline to the peak/dip, which corresponds to the magnitude of $A$ in Eq. (1).


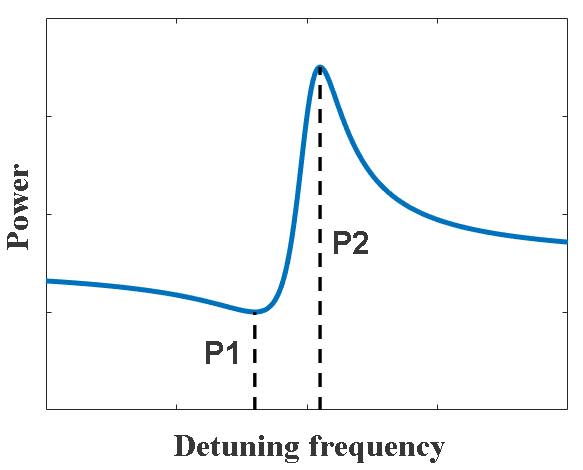


**Fig. S6** Fano profile using the formula in Eq. (2), where $F=1$, $B=2$, and $q=2$. $P1$ is the power at the minimum. which is located at $N=-q$. $P2$ is the power at the maximum, which is located at $N=1/q$.

For the standard Fano line shape (Eq. (2) in the main text), $P_{res}$ in Eq. (2) is the difference between the maximum and minimum power, $P_{res}=|P2-P1|$, as illustrated in Fig. S6. We computed $\nu$ at the minimum and maximum points by taking the 1^st^ derivative of Eq. (2):

|  | $\frac{dI_{SF}}{dN}= \frac{2F(N+q)}{1+N^{2}}\left[ 1-\frac{N(N+q)}{1+N^{2}} \right]$ | (S3) |
| --- | --- | --- |

The 1^st^ derivative is zero at the critical points.

|  | $0= \left( N+q \right)[\left( 1+N^{2} \right)-N(N+q)]$ | (S4) |
| --- | --- | --- |

The two solutions from Eq. (S4) are $N=-q$ and $N=1/q$. $P1$ is the power where N = -q. Substituting this solution into Eq. (2),

|  | $P1= B.$ | (S5) |
| --- | --- | --- |

Substituting $N=1/q$ into Eq. (2),

|  | $P2= \frac{F\left( \frac{1}{q}+q \right)^{2}}{1+\frac{1}{q^{2}}}+B=F\left( 1+q^{2} \right)+B.$ | (S6) |
| --- | --- | --- |

$P_{\mathrm{res}}$ is then derived:

|  | $P_{res}= \left\vert F\left( 1+q^{2} \right) \right\vert.$ | (S7) |
| --- | --- | --- |

The generalized Fano line shape, which is a product of the interaction between two modes and a continuum background^2^, is given by:

|  | $I_{GF}\left( N_{1},N_{2} \right)=B+ I_{1,SF}\left( N_{1} \right)+I_{2,SF}\left( N_{2} \right)=B+ \sum_{i=1}^{2} F_{i}\frac{{(N_{i}+q_{i})}^{2}}{1+{N_{i}}^{2}},$ | (S8) |
| --- | --- | --- |

where $I_{1,SF}\left( N \right)$ and $I_{2,SF}(N)$ are two single Fano resonances described in Eq. (2). This is the derivation of Eq. (3) in the main text.

The fitted parameters from Eq. (S8) are substituted into Eq. (S7) to calculate $P_{res}$ for each mode as given by:

|  | $P_{res, i}=\left\vert F_{i}\left( 1+q_{i}^{2} \right) \right\vert$ | (S9) |
| --- | --- | --- |

The extinction ratio (ER) is calculated by the following equation.

|  | $ER \left[ dB \right]=10\log\left( \frac{Power at peak/dip}{B} \right)$ | (S10) |
| --- | --- | --- |


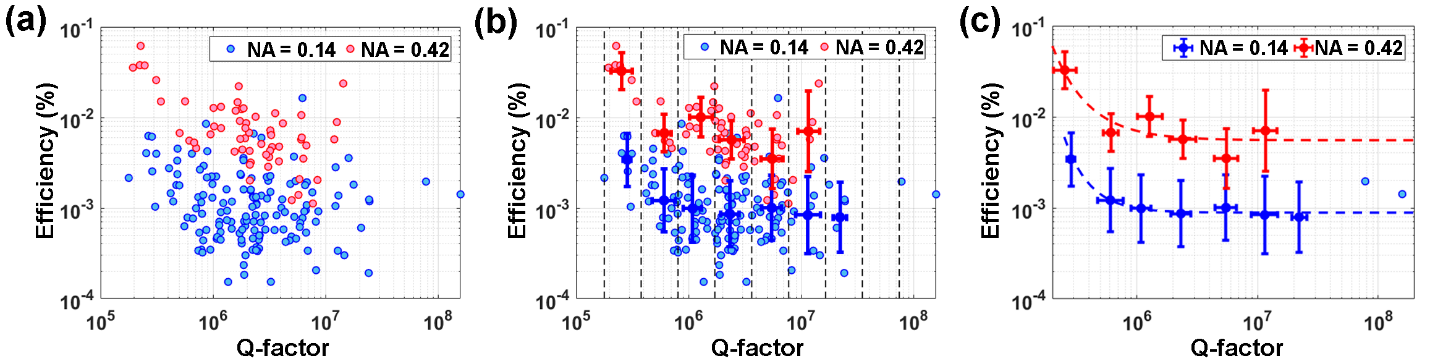


**Fig. S7** Measured resonant power vs Q-factor from two objective lenses: NA of 0.14 and 0.42. **(a)** Raw data. **(b)** Data is divided into 10 groups by Q-factor. Dashed black lines indicate the boundary of each group. Average Q-factor and resonant power are plotted as a single point on each group with standard deviation as an error bar. (c) The experimental data are fitted to curves to guide the eye.

Fig. S7(a) shows the normalized measured resonant power and Q-factor of 140 resonance modes from 57 microtoroids with two different objective lenses: NA of 0.14 and 0.42. To create this plot, we divided the data into ten groups by Q-factor. The dashed black lines indicate the boundary of each group, which is equally separated by Q-factor in log-scale. The average Q-factor and percent efficiency in each group is represented as a single point. Error bars represent the standard deviation in each group as shown by solid lines in Fig. S7(b). $P_{scat}$ captured is limited NA of the microscope objective. To obtain % coupling efficiency, the total scattering power is the product of the scaling factor $\gamma=2\pi/(2\arcsin\mathrm{NA})$ and $P_{scat}$.

**Supplementary Note 4:** Additional coupling map

To observe the modification of line shape as the beam-microtoroid distance changes, a two-dimensional scan in the YZ plane is performed. In addition to Fig. 3, Fig. S8(a-b) shows how the resonance line shape changes when scanning the along the $z$-axis. We defined ($y$, $z$) to be (0,0) at the highest efficiency position. The asymmetric profiles of the changing Fano line shape imply a change in phase difference between two interfering modes.


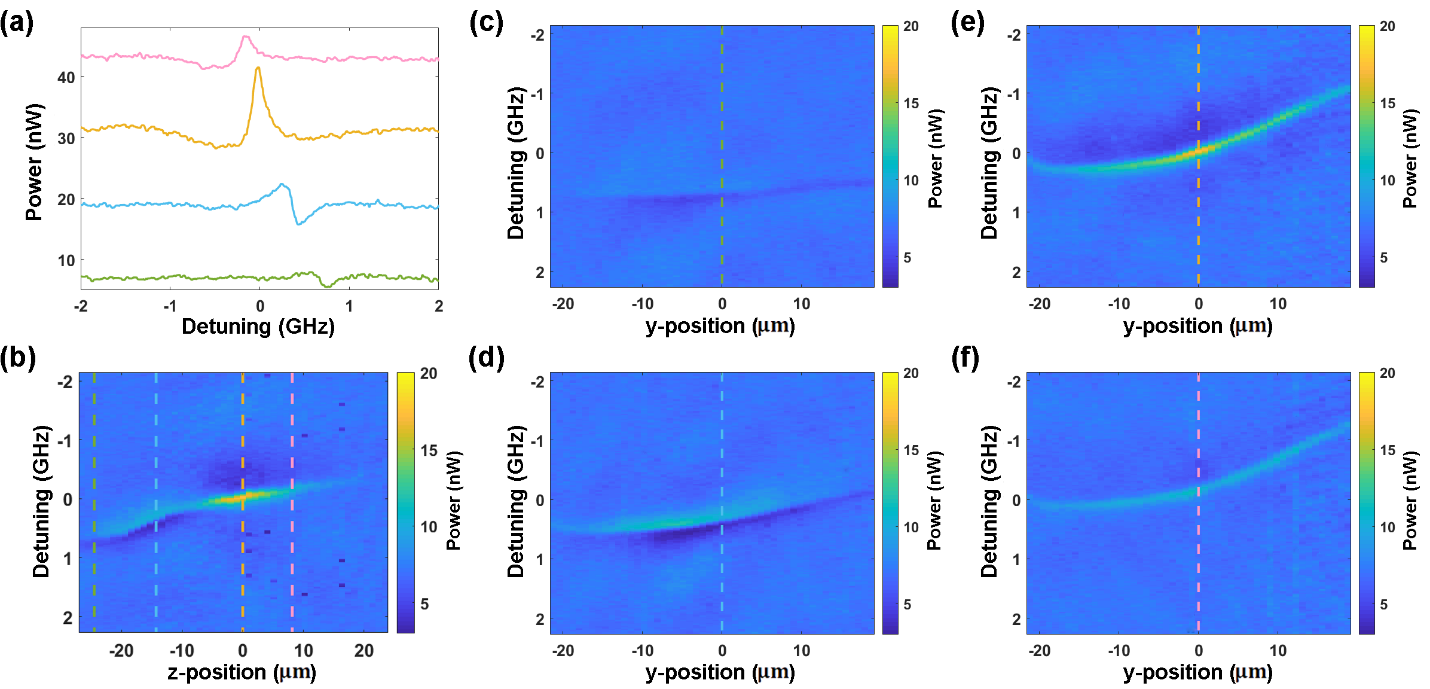


**Fig. S8** Mode spectra using a 5× objective lens (NA = 0.14) at different beam-microtoroid distances. **(a)** From bottom to top: the microtoroid was moved upward. Effectively, the beam was moved downward relative to the cavity. Spectra colors extracted from different z-positons correspond to the mode spectra in (b). **(b)** Mode spectra scanning along the z-axis at y = 0. **(c-f)** Mode spectra scanning along the y-axis at different z-positions. The same color of the dashed line means the same spectra shown in (a) and (b).

In addition to Fig. 5, Fig. S9(a) shows the resonance profile transition at different z-positions. There are at least four modes in this profile. Eq. (S8) was modified to be a summation of four terms to be used for fitting. Since changing the microtoroid position affects the change of effective index differently for different modes, the mode interaction is different at different positions, producing different profiles.


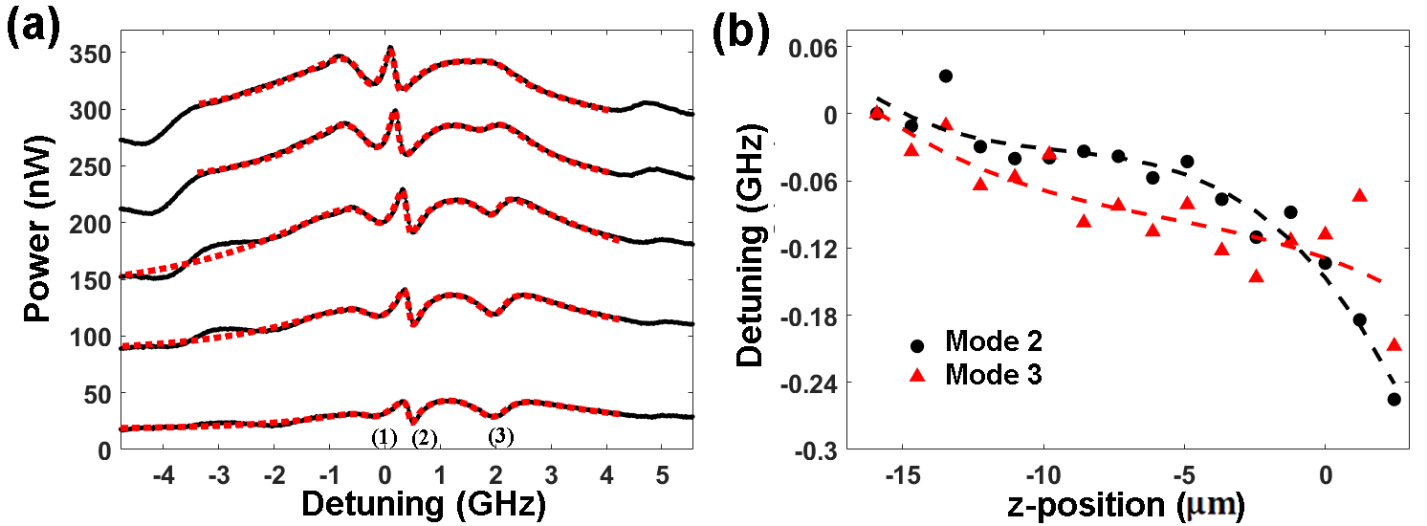


**Fig. S9** Mode spectra using a 5× objective lens (NA = 0.14) at different beam-microtoroid distances. **(a)** From bottom to top: the microtoroid was moved upward. Effectively, the beam was moved downward relative to the cavity. Resonance profiles are fitted with the summation of four modes. The numbers indicate modes. The 4^th^ mode is a low Q-factor mode. **(b)** Resonance shift at different z-positions of two modes: (2) and (3).


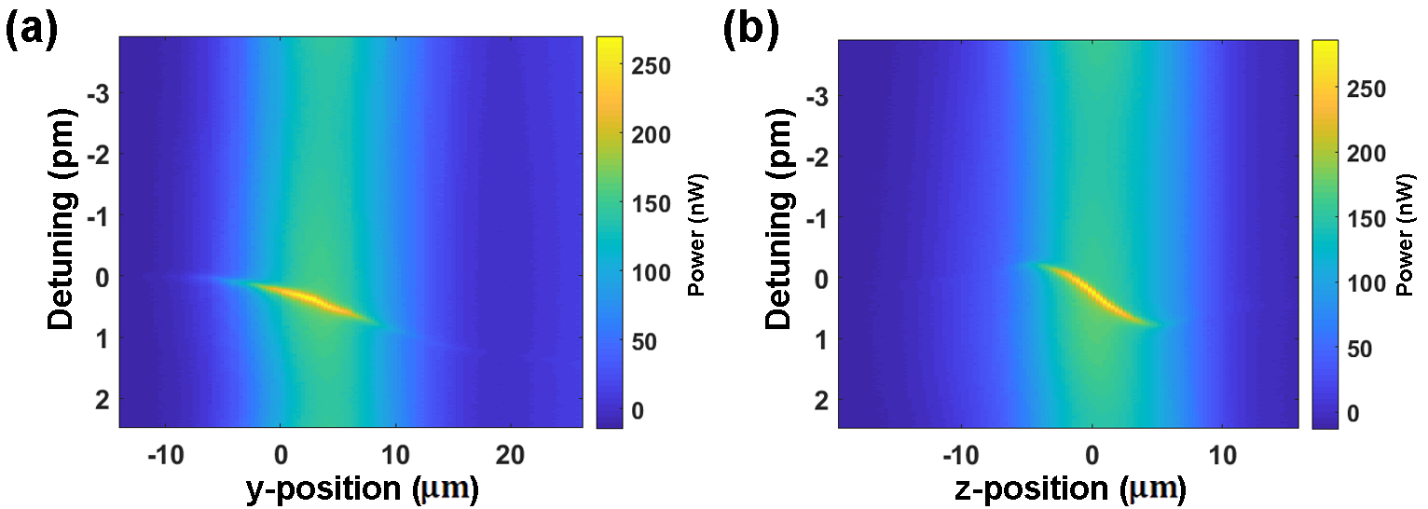


**Fig. S10** Pre-analyzed mode spectra at different positions using a 5x objective lens (NA = 0.14) **(a)** Detuning ($\Delta\lambda$) vs. y-position. **(b)** Detunning ($\Delta\lambda$) vs. z-position

Fig. S10 shows the pre-analyzed data of Fig. 4(c-d). In Fig. S10, it is apparent that when a resonance mode has higher power, the background light is also greater. There are two main sources of background light: resonant scattered light from low Q-modes and stray light. To filter out low Q-modes, we subtracted the average background at each position. This is shown in Fig. 4(c-d).


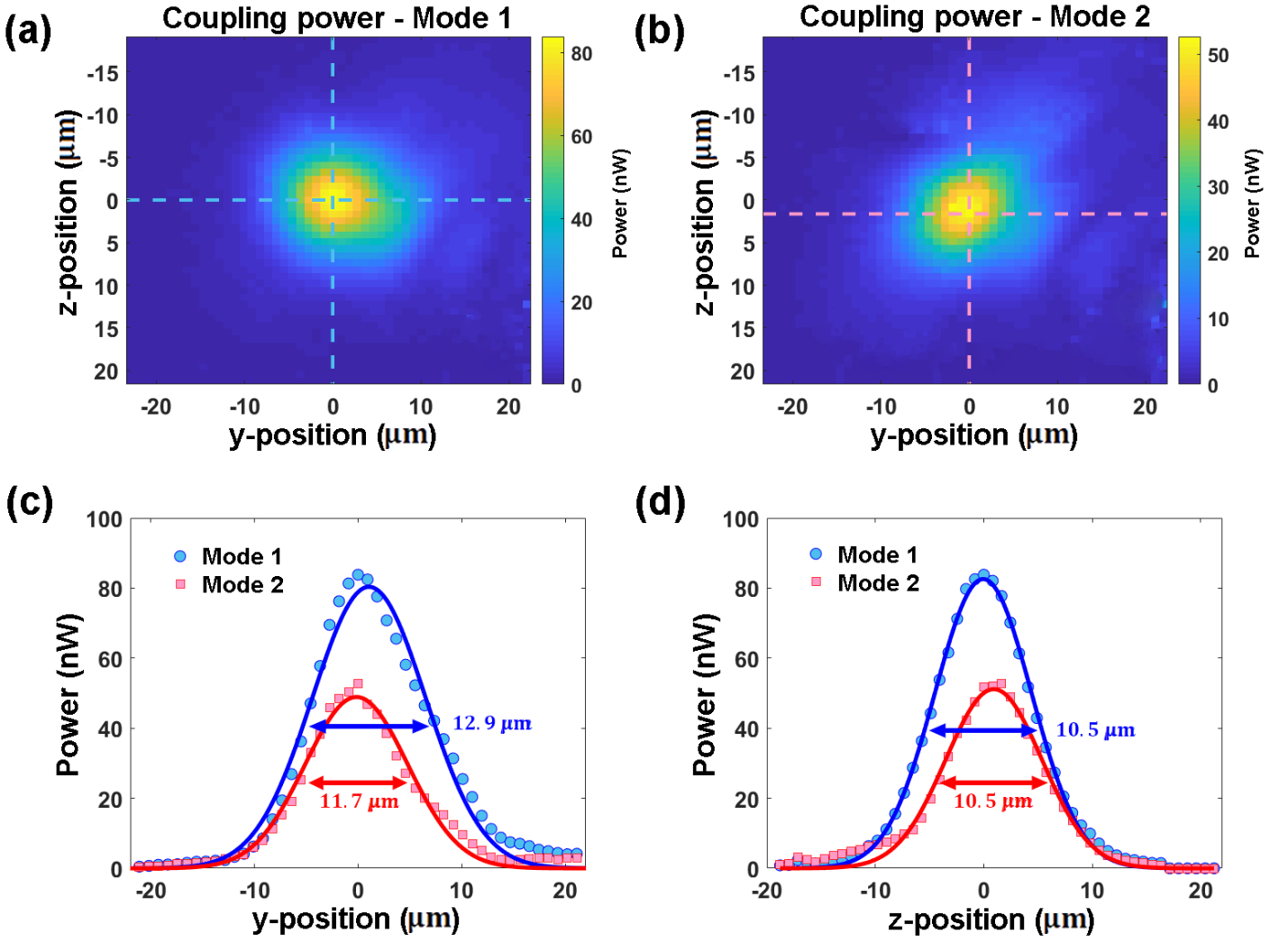


**Fig. S11** Coupling maps of the resonance curve shown in Fig. 5(a): (a) mode 1 and (b) mode 2. (y,z) = (0,0) is defined at the highest power of mode 1. **(a)** Coupling map of mode 1. The dashed blue lines along the y-axis and z-axis cross at (0,0) **(b)** Coupling map of mode 2. The dashed pink lines along the y-axis and z-axis cross at the highest power of mode 2. **(c)** Coupling power along y-axis. Points show power data along the vertical dashed blue line in (a) for mode 1 and power along vertical dashed pink line in (b) for mode 2. **(d)** Coupling power along the z-axis. Points show power data along the horizontal dashed blue line in (a) for mode 1 and power along the horizontal dashed pink line in (b) for mode 2. Solid lines are fit with a Gaussian equation.


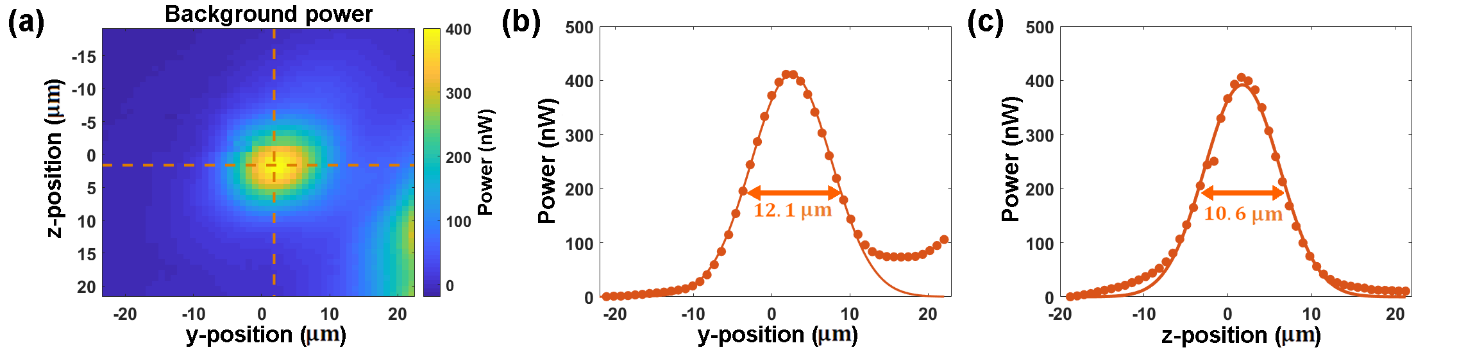


**Fig. S12** Background power map of results in Fig. 5 and Fig. S11. **(a)** Background power map. Dashed orange lines cross at the maximum background power. **(b)** Coupling power along the y-axis. The points show power data along the vertical dashed orange line in (a). **(c)** Coupling power along the z-axis. Points show power data along the horizontal dashed orange line in (a). Solid lines are fits to a Gaussian equation.

In addition to Fig. 5, resonance curves with a generalized Fano line shape were fit with Eq. (S8). Coupling maps of both modes (Fig. S11**(**a-b)) were generated by calculating mode power using Eq. (S9). The maximum power position is slightly different between the two modes. By fitting coupling power along the $y$-axis and $z$-axis to Gaussian equations, the FWHM of both modes, $\sim10 \mu m$, were obtained as shown in Fig. S11(c-d). The background power, Fig. S12, is the $B$ parameter achieved by fitting Eq. (S9) at each position.


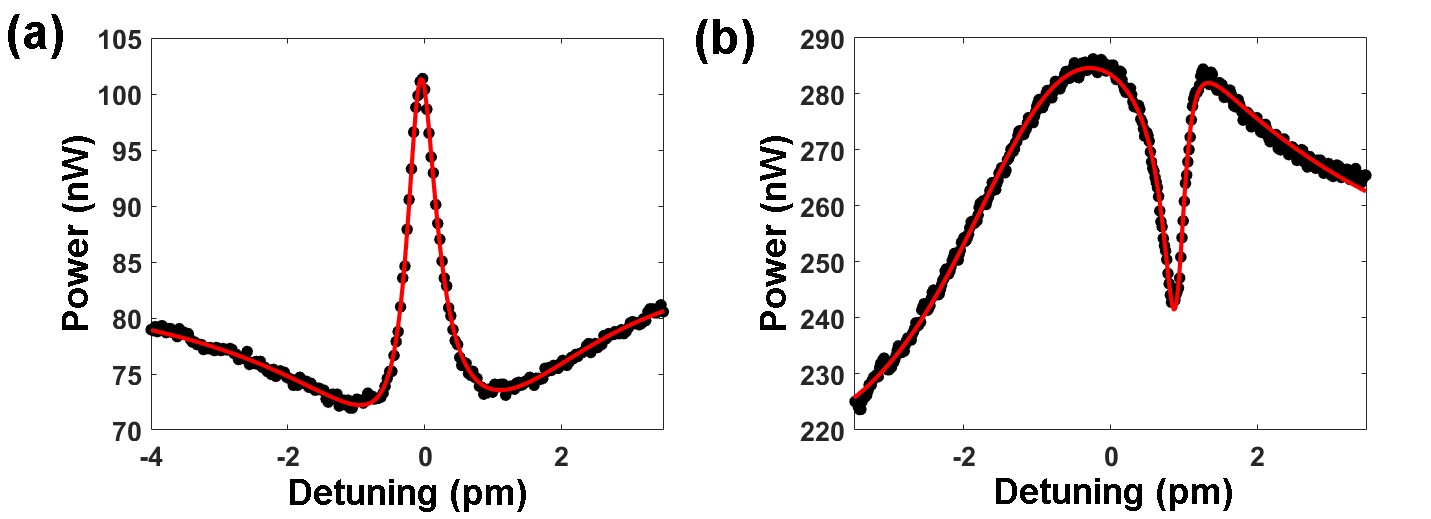


**Fig. S13** Resonance curves using a 20x objective lens (NA = 0.42).

For comparison, we replaced the 5× objective lens with a 20× objective. Fig. S13(a) and (b) show the resonance profiles at (y, z) = (0,0) of Fig. 6(a) and (b), respectively.

**Supplementary Note 5:** Finite element simulation

1. **Electric field distribution**

**
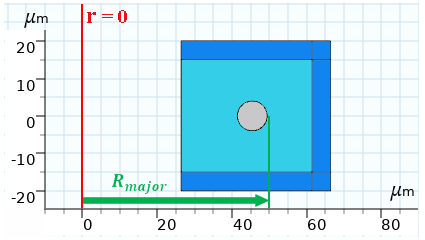
**

**Fig. S14** Finite element simulation geometry for computing electric field in microtoroid cavity. The red line is the azimuthal axis. The grey area is the microtoroid cavity whose refractive index is 1.45. The surrounding blue area is air. The dark blue area is a Perfectly Matched Layer.

A finite element simulation using COMSOL Multiphysics was performed to simulate the electric field in the microtoroid cavity. The system geometry was created in two dimensions around an azimuthal axis as shown in Fig. S14. The simulation was performed by using the Electromagnetic Waves module in the frequency domain. The microtoroid ring, indicated by the grey circle, was made of silica whose refractive index was set to be $n_{SiO_{2}}=1.45$. Based on dimensions obtained from SEM images (see Supplementary Note 1), the major radius ($R_{major}$) and minor radius ($R_{minor}$) were 50 µm and 4 µm, respectively. We studied operation in air. Therefore, the blue and dark blue areas had a refractive index of 1. The dark blue area was defined to be a Perfectly Matched Layer. As an initial guess, the azimuthal mode number was swept $\pm$10 around $\left\lfloor\frac{2\pi R_{major}}{n_{SiO_{2}}} \right\rfloor$with step size of 1, with an approximate eigenfrequency guess of $\frac{c}{n_{SiO_{2}}\lambda}$, where $c$ and $\lambda$ are the speed of light and approximate operating wavelength (780 nm). The azimuthal mode number that yielded the relevant eigenfrequency within the tunable laser range was then selected.

1. **Coupling efficiency**

To simulate free space coupling and the relationship between mode quality factor and coupling efficiency, we generated a 3D COMSOL Simulation using the Wave Optics Module (Fig. S15). The domain includes a straight segment for the incident beam and a curved wedge-shaped segment for a microtoroid with $R_{major}=47$ µm, $R_{minor}=2$ µm, and an adjacent evanescent domain of thickness equal to $\lambda=780$ nm.


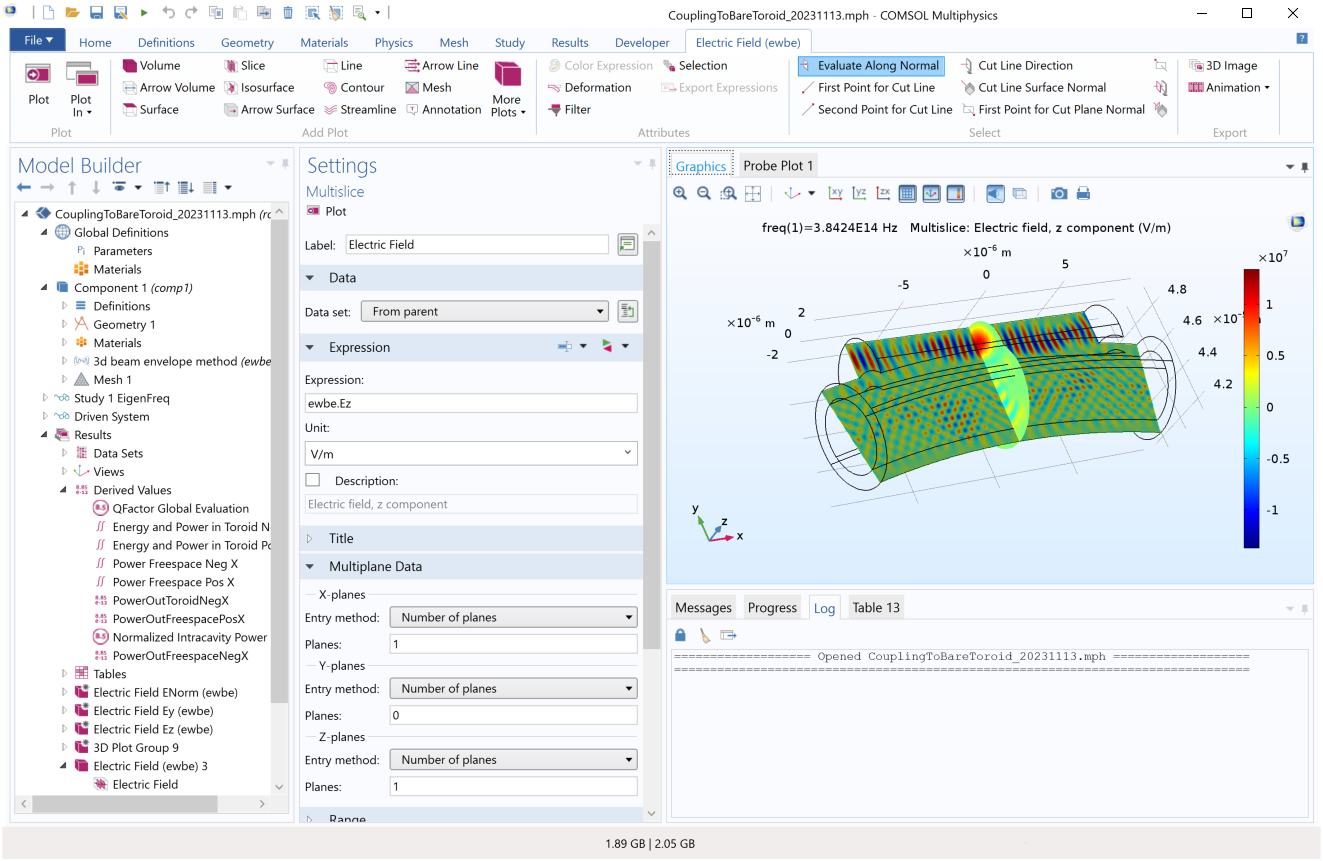


**Fig. S15** 3D COMSOL Simulation for Coupling Efficiency. Light is launched from the top-right of the figure. A wedge section of the toroid is shown in the bottom of the figure. Color indicates the $z$-component of the electric field at one instant in time.

The two flat faces of the straight segment are ports, where the input port has a positive $x$-coordinate, and the output port has a negative $x$-coordinate. The electric field at the input port is that of a propagating Gaussian beam^3^:

|  | $\mathbf{E}\left( r,\zeta\right)=E_{0}{\hat{\mathbf{n}}}_{\mathbf{E}}\frac{w_{0}}{w\left( \zeta\right)}\exp\left( -\frac{r^{2}}{w\left( \zeta\right)^{2}} \right)\exp\left[ -i\left( k\zeta+\frac{kr^{2}}{2R\left( \zeta\right)}-\psi\left( \zeta\right) \right) \right],$ | (S11) |
| --- | --- | --- |

where $r$ is the distance from the optical axis. $\zeta$ is the coordinate along the optical axis, which in this geometry (Fig. S15), is parallel to the $x$-axis, but located at $\left( y,z \right)=(R_{major},0)$. $E_{0}=1$ V m^-1^ is the electric field amplitude. $\hat{n}_{E}$ specifies the polarization of the beam, which we choose to be linear and either parallel to $y$ for coupling to TM modes or parallel to $z$ for coupling to TE modes. $w_{0}=\lambda/(2 \mathrm{NA})$ is the beam waist at $\zeta=0$ and $w\left( \zeta\right)$ is the beam width at a given $\zeta$ coordinate:

|  | $w\left( z \right)=w_{0},\sqrt{1+\left( \frac{\zeta}{\zeta_{R}} \right)^{2}},$ | (S12) |
| --- | --- | --- |

where $\zeta_{R}$ is the Rayleigh range,

|  | $\zeta_{R}=\frac{\pi w_{0}^{2}n_{air}}{\lambda}.$ | (S13) |
| --- | --- | --- |

$R(\zeta)$ is the wavefront curvature, given by,

|  | $\frac{1}{R\left( \zeta\right)}=\frac{\zeta}{\zeta^{2}+\zeta_{R}^{2}}.$ | (S14) |
| --- | --- | --- |

The radius of the straight channel is equal to the beam width at the input port.

The two flat faces of the wedge-shaped microtoroid domain use custom Floquet-wedge boundary conditions^4^ to relate the electric field $\mathbf{E}_{D}$ on the destination boundary to the electric field $\mathbf{E}_{S}$ on the source boundary:

|  | $\left( \begin{matrix} E_{D,x} \\ E_{D,y} \\ E_{D,z} \end{matrix} \right)=\left( \begin{matrix} \cos\phi_{w} & -\sin\phi_{w} & 0 \\ \sin\phi_{w} & \cos\phi_{w} & 0 \\ 0 & 0 & 1 \end{matrix} \right)\left( \begin{matrix} E_{S,x} \\ E_{S,y} \\ E_{S,z} \end{matrix} \right)\exp\left( -i \mathbf{k}_{F}\cdot\left( \mathbf{r}_{D0}-\mathbf{r}_{S0} \right) \right),$ | (S15) |
| --- | --- | --- |

where $\phi_{w}=35.1\times2\pi/m$ is the angle of the wedge being simulated, $\left( \mathbf{r}_{D0}-\mathbf{r}_{S0} \right)={\hat{\mathbf{n}}}_{x} 2 \left( R_{major}-R_{minor} \right)\sin(\phi_{w}/2)$ is the vector between two fixed points on each boundary, and $\mathbf{k}_{F}$ is the Floquet vector, defined as,

|  | $\mathbf{k}_{F}={\hat{\mathbf{n}}}_{x} m \left( 1-\frac{i}{2Q} \right)\left( \frac{2\pi-\phi_{w}}{2\left( R_{major}-R_{minor} \right)\sin\left( \frac{\phi_{w}}{2} \right)} \right).$ | (S16) |
| --- | --- | --- |

The real and imaginary parts of $\mathbf{k}_{F}$ respectively account for the phase and loss incurred by the WGM traveling through the part of the microtoroid that is not in the simulation domain.

All curved boundaries in the simulation use scattering boundary conditions, which act to absorb electromagnetic waves. A tetrahedral mesh is used with element size ranging from 170 nm to 280 nm. The beam envelope method is used to solve the system, which permits larger mesh elements than other simulation methods. The phase of the first wave used by the beam envelope method solver is $m\arctan(y/x)$.

To identify the resonant frequencies, shapes, polarizations, and corresponding azimuthal mode numbers $m$ of a set of high quality modes, 2D axisymmetric simulations (see previous subsection) are first run. Then 3D eigenfrequency simulations are run to identify the same modes in the 3D simulation. Quality factors are calculated from these 3D eigenfrequency simulations by accounting for the loss in the simulation domain together with the loss in the microtoroid outside of the simulation domain,

|  | $Q=\left( \frac{\omega_{0}}{2\Gamma} \right)\left( \frac{2\pi}{\phi_{w}} \right),$ | (S17) |
| --- | --- | --- |

where $\Gamma$ is the damping coefficient returned by COMSOL from the simulation.

Then a driven-system frequency domain simulation is run at the exact eignefrequencies for each mode of interest. The ratio of intracavity power to incident power, $(P_{IC}/P_{input})$ is recorded.

To calculate coupling efficiency, we start from the relationship between resonator finesse $\mathcal{F}$ and cavity loss^5^,

|  | $\mathcal{F=}\frac{\pi}{\alpha\left( \frac{L}{2} \right)-\ln R},$ | (S18) |
| --- | --- | --- |

where $\alpha$ is the power absorption coefficient, $L$ is the round-trip length in the cavity, and $R$ is the mirror reflectivity, which we neglect here, as there are no mirrors in a WGM resonator. In the absence of an input beam, the fractional power loss per round trip would be

|  | $A=1-e^{-\alpha L}.$ | (S19) |
| --- | --- | --- |

So,

|  | $\mathcal{F=}\frac{2\pi}{-\ln\left( 1-A \right)}\approx\frac{2\pi}{A},$ | (S20) |
| --- | --- | --- |

for small $A$.

Finesse is defined as

|  | $\mathcal{F=}\frac{\Delta f_{FSR}}{\Delta f}=\frac{\Delta f_{FSR}Q}{f},$ | (S21) |
| --- | --- | --- |

where $f=mc/(Ln_{\mathrm{eff}})$ is the resonance frequency for azimuthal mode number $m$ with effective refractive index $n_{\mathrm{eff}}$, $\Delta f_{FSR}=c/(Ln_{\mathrm{eff}})$ is the free spectral range, $\Delta f$is the resonance linewidth, and $f$ is the resonance frequency. Then the finesse can be written as $\mathcal{F=}Q/m,$ and, using Eq. (S20), the fractional loss per round trip is $A=2\pi m/Q$.

In steady state, when driven with an external beam the power coupled in equals the power loss, so $(AP_{IC}t_{RT})$ energy is coupled in every round trip, where $t_{RT}$ is the round-trip time. The incident energy in the same time period is $\left( P_{input}t_{RT} \right)$, and coupling efficiency is then calculated by,

|  | $\eta=\frac{AP_{IC}t_{RT}}{P_{input}t_{RT}}\times100\%=\frac{P_{IC}}{P_{input}} \frac{2\pi m}{Q}\times100\%$ | (S22) |
| --- | --- | --- |


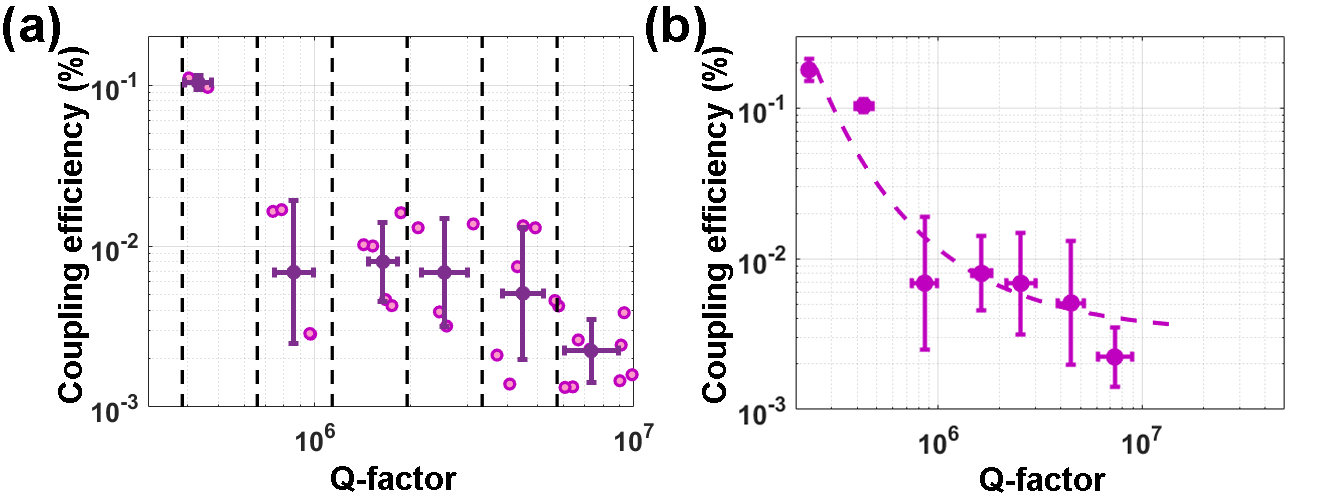


**Fig. S16** Coupling efficiency (%) vs Q-factor **(a)** Simulation data using a 0.42 NA objective lens. Data is divided into 8 groups by Q-factor. Dashed black lines indicate the boundary of each group. Average Q-factor and % coupling efficiency are plotted as a single point on each group with standard deviation as an error bar. **(b)** A curve is fit to the simulation data to guide the eye.

The results of these simulations are plotted in Fig. S16. In the same manner as Fig. S7, the simulation results are divided into eight groups groups by Q-factor. The dashed black lines indicate the interface of each group, which is equally separated by Q-factor in log-scale. The average Q-factor and percent coupling efficiency in each group is represented as a single point. Error bars represent the standard deviation in each group.

**Supplementary Note 6:** Temperature sensing using scanning method

During the temperature sensing experiment, a scan rate of 10 Hz and a scan range of 7 pm were used corresponding to the resolution of $\delta\lambda_{res, scan}\sim46$pm. The strong linearity was observed with a slope of 4.7 pm $℃^{-1}$ as shown in Fig. S17(b).


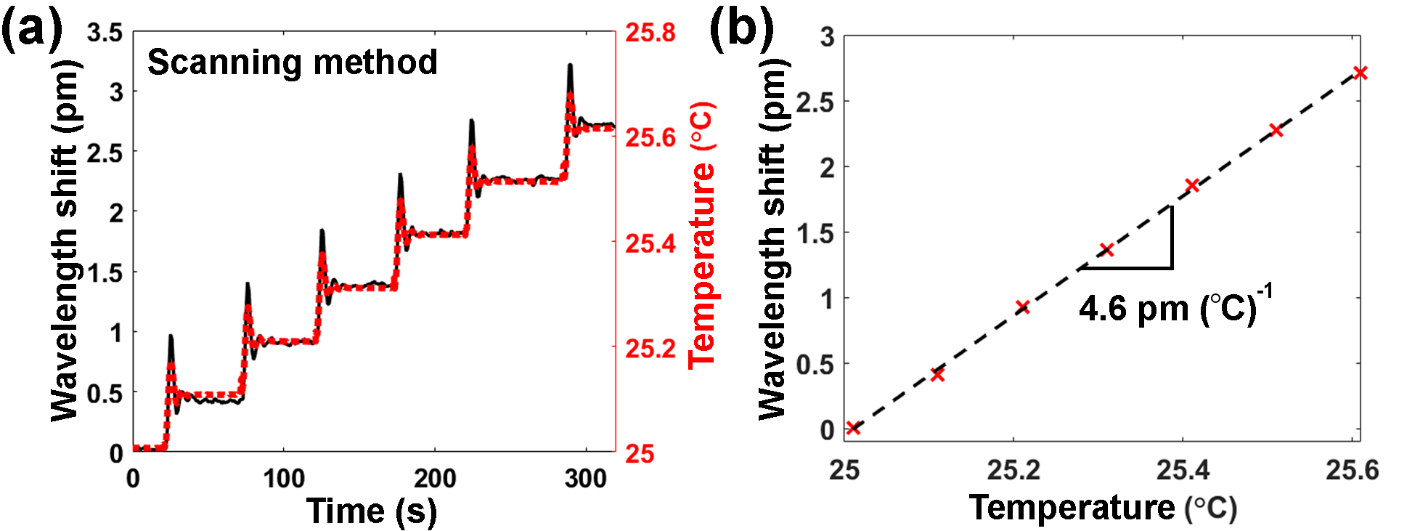


**Fig. S17** (a) Temperature sensorgram using the scanning method. The black and red lines indicate resonance wavelength shift and temperature measured from the thermistor. (b) Resonance waventhlength shift vs temperature. Sensor shows a strong linearity providing a slope of 4.7 pm $℃^{-1}$ .

**Supplementary references**

1. Pongruengkiat, W. & Pechprasarn, S. Whispering-Gallery Mode Resonators for Detecting Cancer. *Sensors* **17**, 2095 (2017).

2. Caselli, N. *et al.* Generalized Fano lineshapes reveal exceptional points in photonic molecules. *Nat Commun* **9**, 396 (2018).

3. Svelto, O. *Principles of Lasers*. (Springer US, 2010). doi:10.1007/978-1-4419-1302-9.

4. Chen, L., Li, C., Liu, Y.-M., Su, J. & McLeod, E. Simulating robust far-field coupling to traveling waves in large three-dimensional nanostructured high-Q microresonators. *Photon. Res.* **7**, 967 (2019).

5. Suter, M. & Dietiker, P. Calculation of the finesse of an ideal Fabry–Perot resonator. *Appl. Opt.* **53**, 7004 (2014).
